# Histone deposition promotes recombination-dependent replication at arrested forks

**DOI:** 10.1371/journal.pgen.1008441

**Published:** 2019-10-04

**Authors:** Julien Hardy, Dingli Dai, Anissia Ait Saada, Ana Teixeira-Silva, Louise Dupoiron, Fatemeh Mojallali, Karine Fréon, Francoise Ochsenbein, Brigitte Hartmann, Sarah Lambert

**Affiliations:** 1 Institut Curie, PSL Research University, UMR3348, Orsay, France; 2 University Paris Sud, Paris-Saclay University, UMR3348, Orsay, France; 3 CNRS, UMR3348, Orsay France; 4 CEA, DRF, SB2SM, Laboratoire de Biologie Structurale et Radiobiologie, Gif-sur-Yvette, France; 5 Laboratoire de Biologie et Pharmacologie Appliquée (LBPA) UMR 8113, CNRS / ENS de Cachan, Cachan cedex, France; MRC Laboratory of Molecular Biology, UNITED KINGDOM

## Abstract

Replication stress poses a serious threat to genome stability. Recombination-Dependent-Replication (RDR) promotes DNA synthesis resumption from arrested forks. Despite the identification of chromatin restoration pathways after DNA repair, crosstalk coupling RDR and chromatin assembly is largely unexplored. The fission yeast Chromatin Assembly Factor-1, CAF-1, is known to promote RDR. Here, we addressed the contribution of histone deposition to RDR. We expressed a mutated histone, H3-H113D, to genetically alter replication-dependent chromatin assembly by destabilizing (H3-H4)_2_ tetramer. We established that DNA synthesis-dependent histone deposition, by CAF-1 and Asf1, promotes RDR by preventing Rqh1-mediated disassembly of joint-molecules. The recombination factor Rad52 promotes CAF-1 binding to sites of recombination-dependent DNA synthesis, indicating that histone deposition occurs downstream Rad52. Histone deposition and Rqh1 activity act synergistically to promote cell resistance to camptothecin, a topoisomerase I inhibitor that induces replication stress. Moreover, histone deposition favors non conservative recombination events occurring spontaneously in the absence of Rqh1, indicating that the stabilization of joint-molecules by histone deposition also occurs independently of Rqh1 activity. These results indicate that histone deposition plays an active role in promoting RDR, a benefit counterbalanced by stabilizing at-risk joint-molecules for genome stability.

## Author summary

DNA replication occurs in the context of DNA packaged into chromatin. At replication fork, the parental chromatin is evicted and re-assembled, together with newly synthetized histones, onto newly replicated DNA thanks to a network of histone chaperones. The progression of replication forks is often interrupted by a plethora of fork obstacles that affect replisome functionality. Such dysfunctional forks are fragile structures prone to chromosomal rearrangements and chromatin changes, leading to human diseases including cancer. Homologous recombination plays a crucial role in the recovery of dysfunctional forks. We have genetically altered the process of histone deposition-coupled to DNA synthesis to interrogate the contribution of chromatin assembly during fork recovery by homologous recombination. We found that histone deposition acts during fork recovery in a manner that recombination structures are protected from disassembly by helicases such as Rqh1. Histone deposition-coupled to fork recovery favors deletion-type recombinant. Chromatin assembly during fork recovery requires only a subset of the histone chaperones normally acting at canonical forks, namely CAF-1 and Asf1. CAF-1 associates to sites of recombination-dependent DNA synthesis during fork recovery. We propose that restarted forks remain coupled to histone deposition, a benefit counterbalanced by the risk to stabilize recombination intermediates detrimental to genome stability.

## Introduction

The maintenance of genome integrity occurs in the context of DNA packaged into chromatin. Chromatin constitutes a barrier to DNA replication and repair machineries that should be first lifted and then restored behind the replication fork or once the repair event is achieved [1]. Genomes are routinely exposed to a variety of DNA damages that induce profound chromatin rearrangements and pose serious threat to epi-genome integrity during DNA replication [2]. Despite the recent identification of chromatin restoration pathways upon DNA repair, the crosstalk and coordination between both processes, that is likely key to safeguard genome integrity, remain poorly understood [3].

The basic unit of chromatin is the nucleosome which consists of 147 bp of double stranded DNA wrapped around a histone octamer containing one (H3-H4)_2_ tetramer and two (H2A-H2B) dimers [4]. During DNA replication, nucleosomes ahead of the replication fork are evicted and both parental and newly synthetized histones are assembled onto newly replicated DNA through a process called replication-coupled chromatin assembly. This process requires a network of chromatin factors that operate sequential reactions to handle histone dynamics at ongoing forks. Nucleosome assembly occurs as a stepwise process in which the (H3-H4)_2_ tetramer is deposited before two (H2A-H2B) dimers [5,6]. Deposition of (H3-H4)_2_ tetramer requires specific histone modifications and H3-H4 chaperones, such as the Chromatin Assembly Factor 1, CAF-1, the Anti-Silencing Factor 1, Asf1, and Rtt106 [7].

CAF-1 plays a key role in nucleosome assembly coupled to DNA synthesis during DNA replication and repair. It associates with the Proliferating Cell Nuclear Antigen (PCNA), the processivity factor of DNA polymerases, to facilitate nucleosome deposition onto DNA *in vitro* [8,9]. CAF-1 is a tri-subunit complex in which the large subunit (human p150, *S*. *cerevisiae* Cac1 and *S*. *pombe* Pcf1), scaffolds interaction with H3-H4 and DNA to allow nucleosome assembly. Recent *in vitro* studies have elucidated how CAF-1 promotes (H3-H4)_2_ tetramer deposition onto DNA [10,11,12,13,14]. One CAF-1 complex binds a single H3-H4 heterodimer, allowing unmasking the C-terminus winged helix domain of p150 to bind DNA. Then, DNA-mediated dimerization of two CAF-1 complexes allows (H3-H4)_2_ tetramer formation and deposition onto DNA. The formation of (H3-H4)_2_ tetramer is necessary to achieve deposition onto DNA and then release H3-H4 from CAF-1. An histone H3 mutant that destabilizes H3-H3’ interface impairs *in vitro* tetramer deposition [12]. Asf1 binds a H3-H4 heterodimer and acts by transferring H3-H4 to CAF-1 and Rtt106 [15]. In yeast models, Asf1 is required for acetylation of H3 at lysine K56 (H3K56Ac), a mark of newly synthetized H3, by the acetyl transferase Rtt109 [16,17]. Also, Asf1 associates with components of the replication machinery and facilitates CAF-1-mediated histone deposition *in vitro* [7,18].

Flaws in the DNA replication process are a source of genome and epi-genome instability. Numerous Replication Fork Barriers (RFBs) and replication-blocking agents interrupt fork elongation, causing recurrent temporary pauses to a single replisome and occasional terminal fork arrest. Stressed forks are fragile DNA structures prone to chromosomal aberrations which may result from faulty replication-based DNA repair events [19]. Chromatin establishment and maturation take place during DNA replication, a critical step for the inheritance of the epigenome [20]. Histone supply and chromatin assembly regulate fork stability and elongation [21,22]. Fork obstacles interfere with histone dynamics, including histone recycling and inheritance of histone marks, resulting in adjacent loci liable to epigenetic changes [2,23]. Thus, stressed forks are instrumental in triggering chromosomal aberrations and chromatin changes by mechanisms that remain to be fully understood.

A variety of DNA repair factors are engaged in the timely resumption of fork elongation. Homologous recombination (HR) is a key DNA repair pathway that preserves fork integrity and replication competence through a process called Recombination-Dependent Replication (RDR) [24]. At the pre-synaptic step, the recombinase Rad51 binds single stranded DNA (ssDNA) exposed at arrested forks, to form a filament with the assistance of mediators such as yeast Rad52 and mammalian BRCA2. After homology search, the Rad51 filament promotes strand invasion into an intact homologous DNA template, usually the sister chromatid or the parental DNA ahead of the fork, to form a displacement loop (D-loop). Then, the invading 3’ end allows DNA synthesis to be primed and the reassembly of replication factors to restart the fork (reviewed in [25]). D-loops can be disassembled by DNA helicases such as the human RecQ helicase BLM and its fission yeast orthologue Rqh1 [26]. Because eukaryotic genomes contain numerous dispersed and repeated sequences, RDR can occasionally generate chromosomal rearrangements. In these circumstances, RecQ helicases are instrumental to limit the likelihood of faulty RDR creating chromosomal aberrations [27,28].

Chromatin factors handle histone dynamics at DNA lesions to provide access to DNA repair machineries and to prime DNA repair [1]. Subsequent to DNA repair, chromatin restoration is a necessary step to engage physiological processes such as transcription restart and turning-off the checkpoint response [29,30]. The crosstalk to couple DNA repair and chromatin restoration are poorly understood and it is unknown if RDR, that allows the resumption of DNA synthesis at dysfunctional forks, is coupled to histone deposition. We and others have reported that HR-mediated DNA synthesis is liable to homology-dependent template switches (TS) during both the initiation of RDR and the progression of restarted forks; those TS being promoted by CAF-1 [27,31,32,33]. We have proposed that CAF-1 prevents the disassembly of the D-loop by Rqh1, in a PCNA-dependent manner [34]. Whether this role of CAF-1 in stabilizing joint-molecules requires its histone deposition activity is unknown.

Here, we advance from our previous work by showing that HR-mediated DNA synthesis is coupled to Asf1 and CAF-1-mediated histone deposition, a step necessary to promote RDR. At a site-specific fork arrest, RDR-coupled histone deposition prevents the disassembly of D-loop intermediates by Rqh1. Consistent with chromatin assembly occurring at specific joint-molecules, CAF-1 recruitment to sites of HR-dependent DNA synthesis requires Rad52. DNA synthesis-coupled histone deposition and Rqh1 act synergistically to promote cell survival to replication stress, but not to DSBs. In the absence of Rqh1, histone deposition favors spontaneous deletion-type recombinants, indicating that the role of chromatin assembly in promoting D-loop stabilization occurs also independently of Rqh1. Our data indicate that nucleosome assembly stabilizes D-loop intermediates to promote replication fork recovery. Therefore, we reveal a novel replication-dependent crosstalk between DNA repair factors and chromatin assembly to ensure repair-synthesis and balance genome stability at sites of fork-arrest.

## Results

### Asf1 promotes TS during initiation of RDR

**Fig 1:**
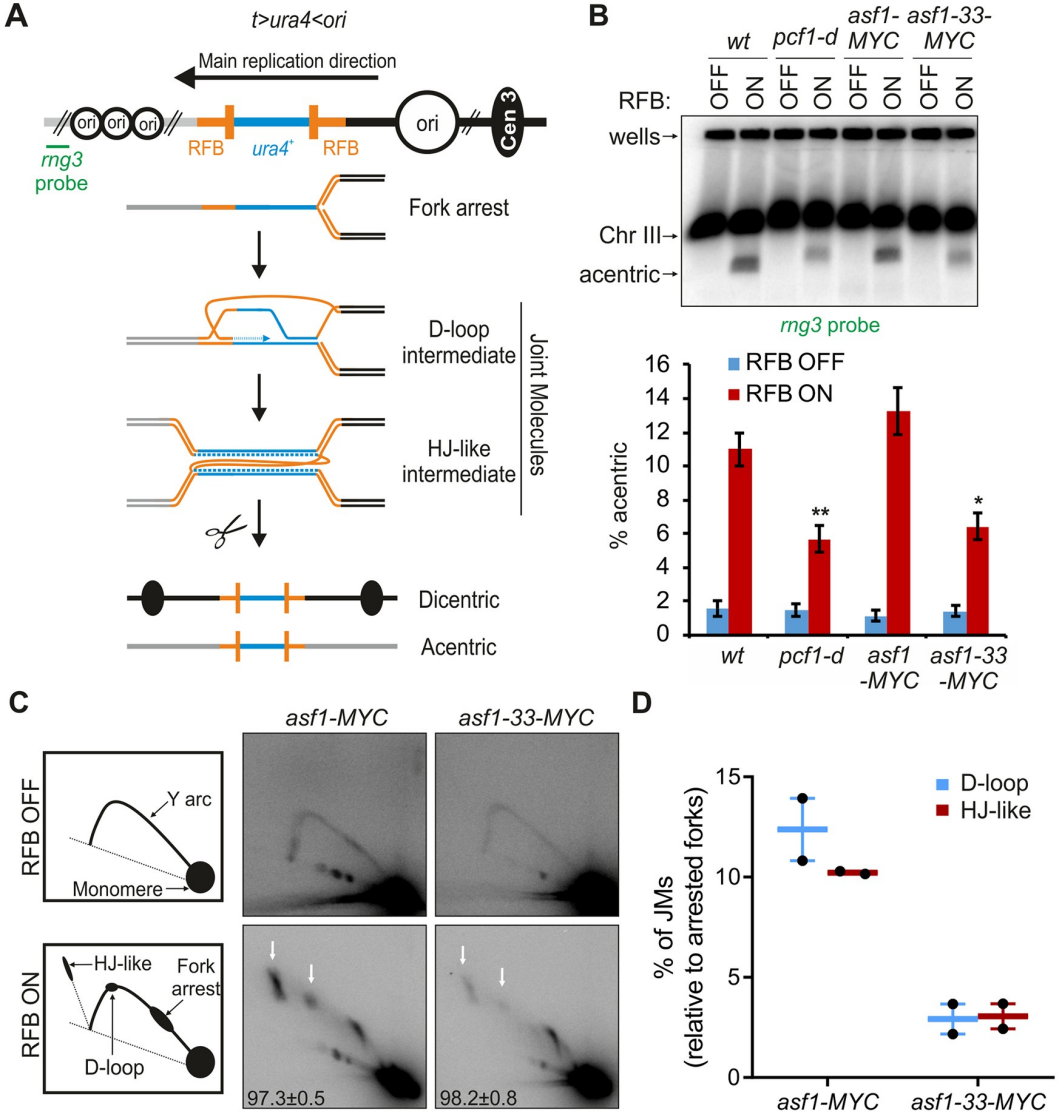
Loss of Asf1 function impairs RDR. **(A)** Diagram of the *t>ura4<ori* locus, in which *t* refers to the telomere-proximal side (gray lines), *ura4* refers to the *wt* gene (blue line), *> and <* refers to the polarity of the two *RTS1*-RFBs (orange bars) and *ori* refers to replication origins (opened black circle, the largest one being the most efficient origin). Green bar indicates the *rng3* probe. The RDR assay consists of a polar Replication Fork Barrier (RFB), called *RTS1*, integrated at the *ura4*^+^ gene, 5Kb away from a strong replication origin at the centromere-proximal side. An inverted repeated *RTS1* sequence is integrated at the telomere-proximal side of *ura4*^+^ to generate the *t>ura4<ori* locus. Due to the main replication direction, the barrier activity of the centromere-proximal *RTS1*-RFB is predominant over the activity of the telomere-proximal RFB. The RFB activity is mediated by the *RTS1*-bound protein Rtf1, the expression of which is regulated by the *nmt41* promoter repressed in the presence of thiamine. Upon Rtf1 expression, > 90% of forks are blocked at the centromere-proximal RFB. The binding of the Rad51 recombinase and its Rad52 loader allow the blocked fork to be restarted to overcome the RFB. Faulty restart events occur in ~ 2–3% of cells/replication: Rad51 promotes newly replicated strands to switch template and to invade the opposite inverted *RTS1* sequence. DNA synthesis is then initiated from the 3’ invading strand on a non-contiguous DNA template, resulting in a stable early JM, referred to as a D-loop intermediate. Upon arrival of the converging fork, a second template switch event results in the formation of a later JM, referred to as Holliday junction (HJ)-like intermediate whose resolution generates at least two recombination products: acentric and dicentric which levels are a marker of RDR completion. **(B)** Top panel: Chromosome analysis in indicated strains and conditions by PFGE and Southern-blot using a radiolabeled *rng3* probe. Bottom panel: Quantification of acentric level normalized to chromosome III level. Values are means of at least 3 independent biological replicates ± standard error of the mean (SEM). Statistical analysis was performed using student’s t-test: * p<0.05, ** p<0.005, compared to *wt*. **(C)** Left panel: schematics of replication intermediates (RIs) observed by 2DGE, in RFB OFF and ON conditions. Right panels: representative 2DGE experiments in indicated strains and conditions. White arrows indicate JMs. Numbers indicate the efficiency of the RFB for each strain analyzed, calculated as the % of arrested fork signal relative to Y arc signal, ± standard deviation (SD) **(D)** Quantification of percentage of JMs levels of panel C relative to replication fork barrier intensity. Individual values are plotted ± the range. See S1 Fig for RDR analysis in additional chromatin factor mutants.

We previously reported that CAF-1 promotes TS during initiation of RDR, in a way that the D-loop is protected from disassembly by Rqh1. We asked if other histone chaperones are involved in RDR. To this end, we employed a previously described site-specific fork arrest assay in which replication of a specific genomic locus is strictly dependent on HR [27]. The assay consists of two polar Replication Fork Barrier (RFB), called *RTS1*, integrated as inverted repeats at both sides of the *ura4*^+^ gene, abbreviated as the *t>ura4<ori* locus (Fig 1A). Upon activation of the RFB, the binding of Rad51 and its Rad52 loader allows blocked forks to be restarted to overcome the RFB. Occasionally, Rad51 promotes newly replicated strands to switch template and invades the opposite *RTS1* sequence to form a D-loop which primes the restarted DNA synthesis on a non-contiguous DNA template. This TS pathway results in the formation of stable joint-molecules (JMs), referred to as D-loop and Holliday junction-like intermediates, whose resolution generates at least two RDR products: an acentric and a dicentric chromosome [34]. The formation of acentric and dicentric chromosome is strictly dependent on Rad52 and serves as a marker for RDR completion [27].

We applied the RDR assay to a temperature-sensitive allele of the essential *asf1* gene, *asf1-33*, which was reported to be defective for H3 deposition at restrictive temperature [35]. Cells were cultured at semi-permissive temperature (32°C) at which *asf1-33* mutated cells exhibited sensitivity to 0.02% methyl methane sulfonate (MMS, an alkylated DNA agent that impedes replication fork progression) (S1A Fig). Chromosome analysis by Pulsed Field Gel Electrophoresis (PFGE) coupled with Southern-blot hybridization showed that the amount of acentric fragment, a RDR product, was reduced by 2 fold in *asf1-33* cells, as well as in a mutant lacking CAF-1 (*i*.*e*. in *pcf1* deleted cells), indicating that Asf1 promotes RDR (Fig 1B). The analysis of replication intermediates by two-dimensional gel electrophoresis (2DGE) showed that signals corresponding to arrested forks were similar in all analyzed strains (see bottom left numbers on Fig 1C), indicating that the defect in RDR is not a consequence of a less efficient RFB activity in strains lacking Asf1 (Fig 1C). JMs intensity was reduced in the *asf1-33* mutant (Fig 1C and 1D), suggesting that Asf1 preserves JMs during RDR. We concluded that Asf1 promotes TS during initiation of RDR, as reported for CAF-1.

We investigated the role of other histone chaperones in RDR by analyzing the level of acentric chromosome upon RFB induction. We found no decrease on acentric level in cells lacking the HIRA complex (involved in replication-independent H3-H4 deposition), the FACT complex, Rtt106, the two orthologues of the *S*. *cerevisiae* H2A-H2B histone chaperone Nap1 and Nap2, and Chz1, a histone variant H2AZ chaperone (S1B and S1C Fig). Of note, we observed a slight increase in the level of the acentric chromosome in the absence of Nap1, but this was not observed in the absence of the other H2A-H2B histone chaperone Nap2. Thus, the two key histone chaperones Asf1 and CAF-1, known to mediate (H3-H4)_2_ tetramer deposition in a DNA-synthesis dependent manner, promote RDR.

### The mutated histone H3-H113D disrupts (H3-H4)_2_ tetramer formation

To address the role of histone deposition during RDR, we decided to genetically disrupt replication-dependent chromatin assembly by altering the stability of (H3-H4)_2_ tetramer to inhibit their stable deposition onto DNA. To this end, we employed a mutated form of H3 containing a single substitution of histidine to aspartic acid (H3-H113D). This mutation was reported to inhibit CAF-1-mediated nucleosome deposition *in vitro* [36].

**Fig 2:**
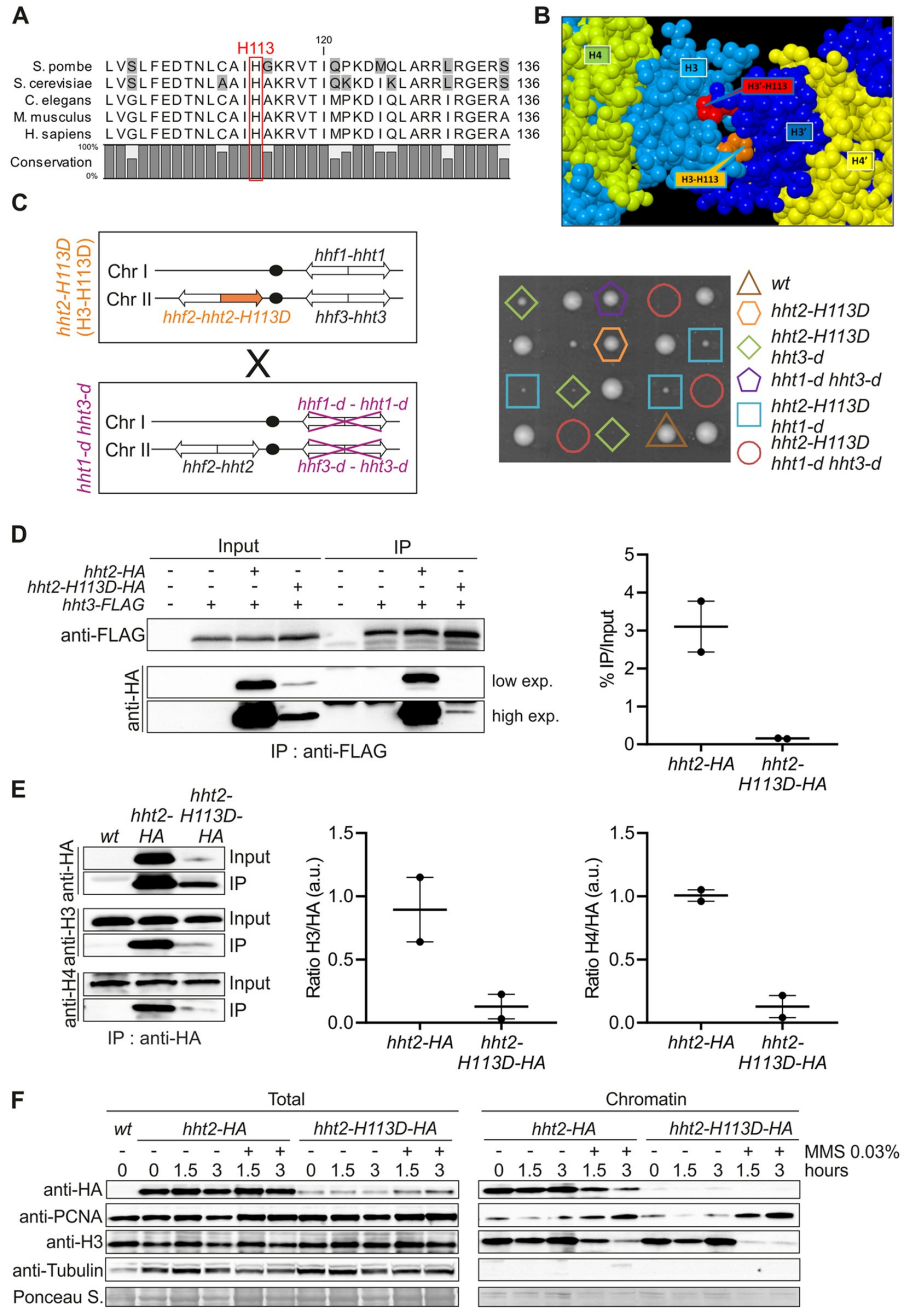
The mutated histone H3-H113D destabilizes (H3-H4)_2_ tetramer. **(A)** Alignment of yeast, worm, mouse and human histone H3. Red rectangle indicates the position of histidine 113. UniProtKB access codes used are *S*. *pombe*: P09988; *S*. *cerevisiae*: P61830; *C*.*elegans*: P08898; *M*. *musculus*: P84228 and *H*. *sapiens*: Q71DI3. **(B)** View of the H3:H3’ interface. The interface between the two histones H3, colored here in light (H3) and dark blue (H3’), maintains H3 and H4 in the tetramer form (H3-H4)_2_. In the H3:H3’ interface, H113 of each histone (orange for H113 in H3 and red for H113 in H3’) is deeply buried in the adjacent histone partner. For clarity, the other histones H2A and H2B, as well as DNA, are not represented. **(C)** Left panel: schematic of the strains crossed. Right panel: spore viability analysis of indicated genotypes. **(D)** Left panel: association of H3-FLAG with H3-HA and H3-H113D-HA in indicated strains. Right panel: quantification expressed in arbitrary unit (a.u.). Individual values are plotted ± the range. **(E)** Left panel: association of H3-HA and H3-H113D-HA with untagged H3 and H4 in indicated strains. Right panels: quantification expressed in arbitrary unit (a.u.). Individual values are plotted ± the range. **(F)** Chromatin association of analyzed proteins in indicated strains and conditions (hours upon MMS addition or not). Two independent biological replicates were performed and representative blots are shown. See S2 Fig for structural impact of H3-H113D.

The interface between the two H3-H4 dimers involves the C-terminal region (residues 106 to 131) of the two histones H3, called here H3 and H3’ [4,37] (Fig 2A and 2B and S2A Fig). We examined X-ray structures of nucleosomes, chosen among the numerous available experimental models on the basis of homology with *S*. *pombe* histone H3 (see details in Materials and methods). The H113 emerges as a key residue in the H3:H3’ interface with H113 of one histone H3 being anchored to the second histone H3’ by a dense network of contacts involving six residues (S2B Fig). Each H113 forms two intermolecular hydrogen bonds with C110 and D123, reinforced by Van der Waals contacts with four residues: A114, R116, K122 and L126. By replacing a neutral or positively charged histidine residue by a negatively charged aspartate that is positioned in front to another aspartate, D123, the H113D mutation generates a prohibitive electrostatic repulsion in the intact H3:H3’ organization. Previous works reported that several mutations (C110E, L12R-I130R, H113A, L126A and A114Y) prevent the H3:H3’ interface formation because of an overwhelming energetic penalty; such mutations are lethal in budding yeast [12,38,39]. By analogy with these cases, the H113D mutation likely drastically destabilizes the H3:H3' interface, precluding the (H3-H4)_2_ tetramer formation.

In *S*. *pombe* cells, 3 genes (*hht1*, *hht2* and *hht3*) encode a single histone H3 protein. The H113D mutation was introduced in the *hht2* gene, which is expressed throughout the cell cycle [40,41]. Cells expressing H3-H113D were viable with no apparent growth defect (Fig 2C). The deletion of *hht1* and *hht3* is viable (*hht1-d hht3-d*) and cells maintain histone H3 protein levels similar to those in *wild type* (*wt*) strain [42,43]. To obtain a strain expressing only H3-H113D, the *hht2-H113D* mutated strain was crossed with an *hht1-d hht3-d* strain. Combining the *hht2-H113D* mutation with both deletions resulted in a synthetic lethality (Fig 2C). Thus, when H3-H113D is the sole histone H3 expressed, cells are not viable, in agreement with this mutation inhibiting (H3-H4)_2_ tetramer formation. Still, we observed that combining *hht2-H113D* with either single *htt1* or *hht3* deletion preserves cell viability but causes a severe synthetic growth defect (Fig 2C). This strongly suggests that *wt* H3 is abundant enough to allow *wt* (H3-H4)_2_ tetramer to be formed and to preserve cell viability but mixed (H3-H113D-H4-H3-H4) tetramer are also unstable. In further experiments, we have employed *hht2-H113D* mutation in *hht1^+^ hht3^+^* background, unless stated otherwise.

We probed H3-H113D associations with histone H3 and H4 in *hht2-H113D* mutated cells (*hht1*^+^ *hht3*^+^). The *hht3* gene was fused to the FLAG epitope and *hht2* or *hht2-H113D* was fused to the HA epitope. Although, the protein level of H3-H113D-HA was lower than the one of *wt* H3-HA (Fig 2D and 2E and S2C Fig), immuno-precipitation experiments clearly showed that H3-FLAG association with H3-H113D-HA was severely reduced, compared to H3-HA (Fig 2D). Reciprocally, H3-H113D-HA association with *wt* H3 and H4 were severely reduced, compared to H3-HA (Fig 2E). These data indicate that mixed (H3-H113D-H4-H3-H4) tetramers are highly unstable. Of note, a similar reduced expression level was observed when the H113D mutation was introduced in *hht3* and *hht1* genes (S2C Fig).

We asked if H3-H113D is incorporated into chromatin using chromatin fraction assays. As expected, H3-HA was found chromatin-bound whereas H3-H113D-HA was not detected in the chromatin fraction, indicating that this mutated histone is very poorly incorporated into nucleosomes assembled onto DNA (Fig 2F). The *wt* H3 (expressed from *hht1* and *hht3* gene) was equally detected in the chromatin fraction of *hht2-HA* and *hht2-H113D-HA* cells, indicating that *wt* H3 is *in fine* incorporated within *wt* nucleosomes in H3-H113D expressing cells. MMS-induced DNA damage resulted in a slightly more abundance of PCNA bound to the chromatin whereas H3-H113D-HA was still not detected in the chromatin fraction. Our data indicate that in *hht2-H113D* mutated cells, H3-H113D inhibits stable tetramer formation and is therefore not deposited onto chromatin, while *wt* (H3-H4)_2_ tetramers are formed and assembled onto chromatin.

### H3-H113D alters the process of replication-coupled nucleosome assembly

**Fig 3:**
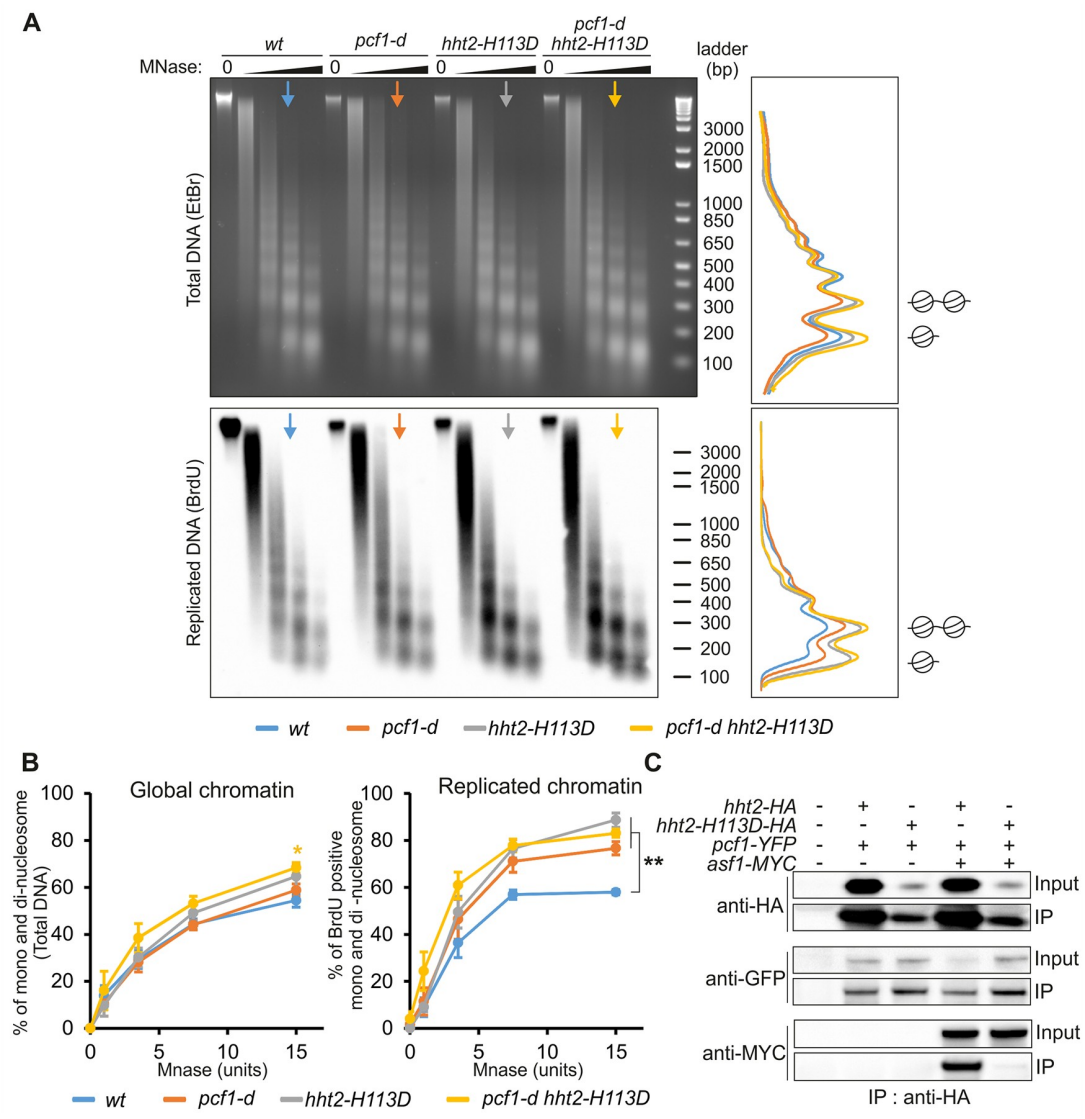
H3-H113D alters replication-coupled chromatin assembly. **(A)** Nucleosomal organization in indicated strains by analysis of MNase-digestion (0, 1, 3, 7.5 and 15 units) patterns of BrdU-incorporated genomic DNA. Total DNA (top) and corresponding BrdU-labelled replicated DNA (bottom) are shown. Densitometric profiles of the 7.5U MNase digestion product (arrows) of indicated strains are shown (left panels). Positions of the oligonucleosomes are indicated (mono and di nucleosomes). **(B)** Percentage of mono and di-nucleosome for the global chromatin (relative to total EtBr signal, left panel) and BrdU-positive mono and di-nucleosome (relative to total BrdU signal, right panel) according to the MNase units. Values are means of at least 3 independent biological replicates ± SEM. Statistical analysis was performed using student’s t-test: * p<0.05; **p<0.005, compared to *wt*. The yellow star on the left panel corresponds to the p value between *wt* and *pcf1-d hht2-H113D*. **(C)** Association of H3-HA and H3-H113D-HA with Pcf1-YFP and Asf1-MYC in indicated strains. See S3 Fig for BrdU incorporation and S4 Fig for protein-protein interactions.

We investigated the consequences of inhibiting tetramer formation on the chromatin structure *in vivo*. We asked whether nucleosome organization was perturbed in cells expressing H3-H113D by examining the nucleosomal status of total DNA and newly replicated DNA by micrococcal nuclease (MNase) digestion. We employed strains able to incorporate BrdU, a thymidine analogue [44]. Cells were blocked in early S-phase by hydroxyurea treatment and released in BrdU-containing media for 20 minutes to label newly replicated DNA. BrdU incorporation was detected in MNase-digested chromatin only when cells were released from HU block, showing that BrdU-labelling marks the replicated chromatin (S3 Fig). Upon increased amount of MNase, clear nucleosomal ladders were detectable for total DNA in all strains analyzed (Fig 3A, top panel, EtBr staining). The quantification of mono and di-nucleosome showed that the global chromatin was not more sensitive to MNase in the absence of CAF-1 (*i*.*e*. in *pcf1-d* cells) or in cells expressing H3-H113D (Fig 3B, left panel, Global chromatin). A slight increase in MNase sensitivity was observed in the *pcf1-d hht2-H113D* mutant compared to *wt* cells (p<0.05 yellow star on Fig 3B, left panel). In contrast and consistent with reports in other organisms [45,46,47], the absence of CAF-1 resulted in an alteration of the nucleosomal organization of newly replicated DNA (Fig 3A, bottom panel, BrdU staining). Compared to *wt* strain, the proportion of BrdU-positive mono and di-nucleosome population was significantly increased in *pcf1-d* cells, revealing a higher sensitivity to MNase as an indication of a decreased nucleosomal density of the newly replicated chromatin (Fig 3B, right panel, Replicated chromatin, p<0.005). This is consistent with the loss of the chromatin assembly function of CAF-1. A nucleosomal ladder was still detected in *pcf1-d* cells, indicating that, *in vivo*, nucleosome assembly on newly replicated DNA can occur to some extent in the absence of CAF-1. In human cells, the histone chaperone HIRA acts as a salvage pathway to maintain chromatin integrity during DNA replication [48]. Consistent with this, we found that the deletion of *pcf1* is synthetic lethal with the deletion of *hip1*, the gene encoding one subunit of the fission yeast HIRA complex (S4A Fig). Therefore, the highest MNase sensitivity of the newly replicated DNA observed in *pcf1-d* cells is consistent with a transient alteration of the replication-coupled chromatin assembly process. Remarkably, we observed a similar increased in MNase sensitivity of the newly replicated chromatin in cells expressing H3-H113D (Fig 3A and 3B, right panel, p<0.005), indicating that replication-coupled chromatin assembly is altered in these cells. The nucleosomal pattern of the newly replicated DNA was similarly affected in the double mutant *pcf1-d hht2-H113D*, compared to each single mutant, suggesting that the absence of CAF-1 and unstable (H3-H4)_2_ tetramers alter the same replication-coupled chromatin assembly pathway (Fig 3A and 3B, right panel, p<0.005). These data are consistent with the report that H3-H113D inhibits *in vitro* the chromatin assembly activity of CAF-1 [36]. Together with the observation that H3-H113D is poorly incorporated into assembled nucleosome onto DNA without affecting the MNase sensitivity of total DNA, our data are consistent with a transient defect in replication fork-associated chromatin assembly in cells expressing H3-H113D, as observed in the absence of CAF-1. Although other histone deposition pathways might be affected, our data support that the CAF-1-mediated chromatin assembly pathway is impaired *in vivo* in H3-H113D expressing cells.

We probed for interactions between H3-H113D and histone chaperones involved in RDR. We found that H3-H113D-HA was poorly bound to Asf1-MYC, compared to H3-HA (Fig 3C). However, the binding of Asf1-MYC to *wt* H3 was not affected in cells expressing H3-H113D, arguing that the histone chaperone function of Asf1 is preserved in *hht2-H113D* mutated cells (S4B Fig). Then, we employed a strain expressing Pcf1-YFP that we previously showed targeted to replication foci and able to interact with PCNA [34]. Unexpectedly, we found that *pcf1-YFP* was synthetic lethal with the deletion of *hip1*, as observed for the deletion of *pcf1* (S4A Fig). Nonetheless, Pcf1-YFP was able to interact with H3, H4, PCNA and Pcf2-MYC (S4C–S4E Fig). Thus, Pcf1-YFP is able to promote protein-protein interactions essential to its chromatin assembly function, but it is not functional enough to ensure cell viability in the absence of HIRA. Then, we probed for interactions with H3-H113D and found that Pcf1-YFP bound H3-H113D-HA to the same extent than H3-HA (Fig 3C and S4E Fig). Also, in cells expressing H3-H113D, the ability of Pcf1-YFP to bind Pcf2-MYC, PCNA and H3 and H4 was not affected (S4C–S4E Fig). Altogether, these data indicate that in *hht2-H113D* mutated cells, CAF-1 forms complexes with H3-H4, H3-H113D, and PCNA. Since H3-H113D inhibits tetramer formation, a step necessary to CAF-1 promoting histone deposition, our data are consistent with H3-H113D altering CAF-1 function in replication-coupled chromatin assembly *in vivo*, as observed *in vitro* [36].

### Histone deposition promotes TS during initiation of RDR by preventing D-loop disassembly

**Fig 4:**
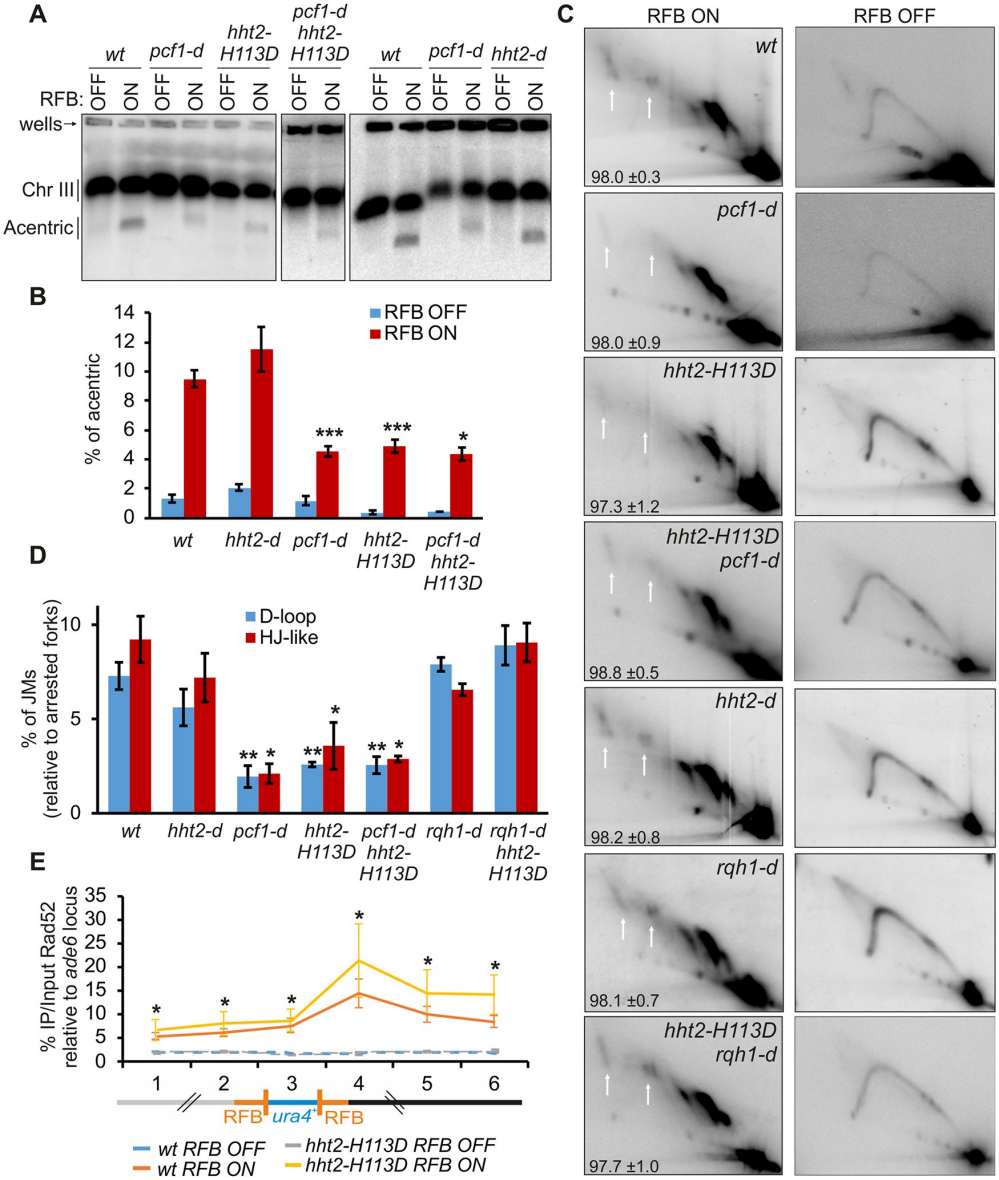
Histone deposition promotes TS during the initiation of RDR. **(A)** Chromosome analysis in indicated strains and conditions as described on Fig 1. **(B)** Quantification of acentric level as described on Fig 1. Values are means of at least 3 independent biological replicates ±SEM. Statistical analysis was performed using student’s t-test: * p<0.05, *** p<0.0005, compared to *wt*. **(C)** Representative 2DGE analysis in indicated strains. White arrows indicate JMs. Numbers indicate the efficiency of the RFB for each strain analyzed, calculated as the % of arrested fork signal relative to Y arc signal ± SD **(D)** Quantification of percentage of joint-molecule levels of panel C relative to replication fork barrier intensity. Values are means of at least 3 independent biological replicates ±SEM. Statistical analysis was performed using student’s t-test: * p<0.05, ** p<0.005, compared to *wt*. **(E)** Rad52 binding to the *t>ura4<ori* locus in RFB OFF and ON conditions. Schematic at the bottom depicts the primers location within the *t>ura4<ori* locus: primer pairs 1 and 2 are located 400 and 200 bp away from the telomere-proximal RFB, respectively; primer pair 3 is within the *ura4* gene; primer pairs 4, 5 and 6 are located 110, 450, and 630 bp away from the centromere-proximal RFB. Values are means of 4 independent biological replicates ±SEM. Statistical analysis was performed using Mann and Whitney U test: * p<0.05. See S5 Fig for the role of H3K56Ac in RDR.

The H3-H113D mutation offers the possibility to question the role of replication-coupled chromatin assembly in promoting TS during the initiation of RDR. We thus applied the RDR assay to *hht2-H113D hht1*^+^ *hht3*^+^ mutated cells and found that JMs intensity was reduced as well as the subsequent accumulation of acentric chromosome, one RDR product (Fig 4A–4D). Of note, the efficiency of the RFB was found similar in all strains analyzed (see bottom left numbers on Fig 4C). Rad52 was able to bind the active RFB in cells expressing H3-H113D, indicating that H3-H113D impairs RDR downstream Rad52 recruitment to arrested forks, as reported for CAF-1 (Fig 4E) [34]. Importantly, similar defects in RDR were observed in the double *pcf1-d hht2-H113D* mutant compared to each single mutant, showing that H3-H113D impairs RDR by altering CAF-1-mediated histone deposition. We reported that in the absence of CAF-1, JMs are faster disassembled by Rqh1 [34]. Remarkably, similar interactions were observed in *hht2-H113D* cells in which the deletion of *rqh1* restored the intensity of JMs signal, whereas the deletion of *rqh1* alone did not affect the intensity of JMs (Fig 4C and 4D). No defect in RDR were observed in strains in which genes encoded H3 are deleted (either *hht2-d* or *hht1-d hht3-d* cells (Fig 4A–4D and S5 Fig), further supporting that H3-H113D favors D-loop disassembly as a consequence of impairing replication-coupled histone deposition, not as a consequence of its lower abundance. Altogether, these data establish that histone deposition promotes TS during RDR by preventing D-loop disassembly by Rqh1.

### H3K56Ac is dispensable to TS during initiation of RDR

The H3K56Ac modification marks newly synthetized histone and was proposed to regulate nucleosome assembly to contribute to the DNA damage response [49]. Hence, the importance of acetylation in addition to histone deposition has to be considered. We found that in contrast to *wt* H3 that was efficiently acetylated by Rtt109 in *hht2-H113D* cells, we could no detect the K56 acetylation of the mutated H3-H113D form (S5A Fig), consistent with H3-H113D being unable to associate with Asf1-MYC (Fig 3C). We thus tested the role of H3K56Ac in RDR. We analyzed two strains: one expressing a single H3 protein which cannot be acetylated on K56 (*hht1-d hht3-d hht2-K56R*) and a *rtt109* deleted strain. Consistent with previous reports [17], both strains were defective for H3K56Ac (S5B Fig). We observed no changes on the level of acentric chromosome (S5C and S5D Fig). Thus, H3K56Ac is dispensable to TS during the initiation of RDR.

### CAF-1 recruitment to sites of HR-dependent DNA synthesis

**Fig 5:**
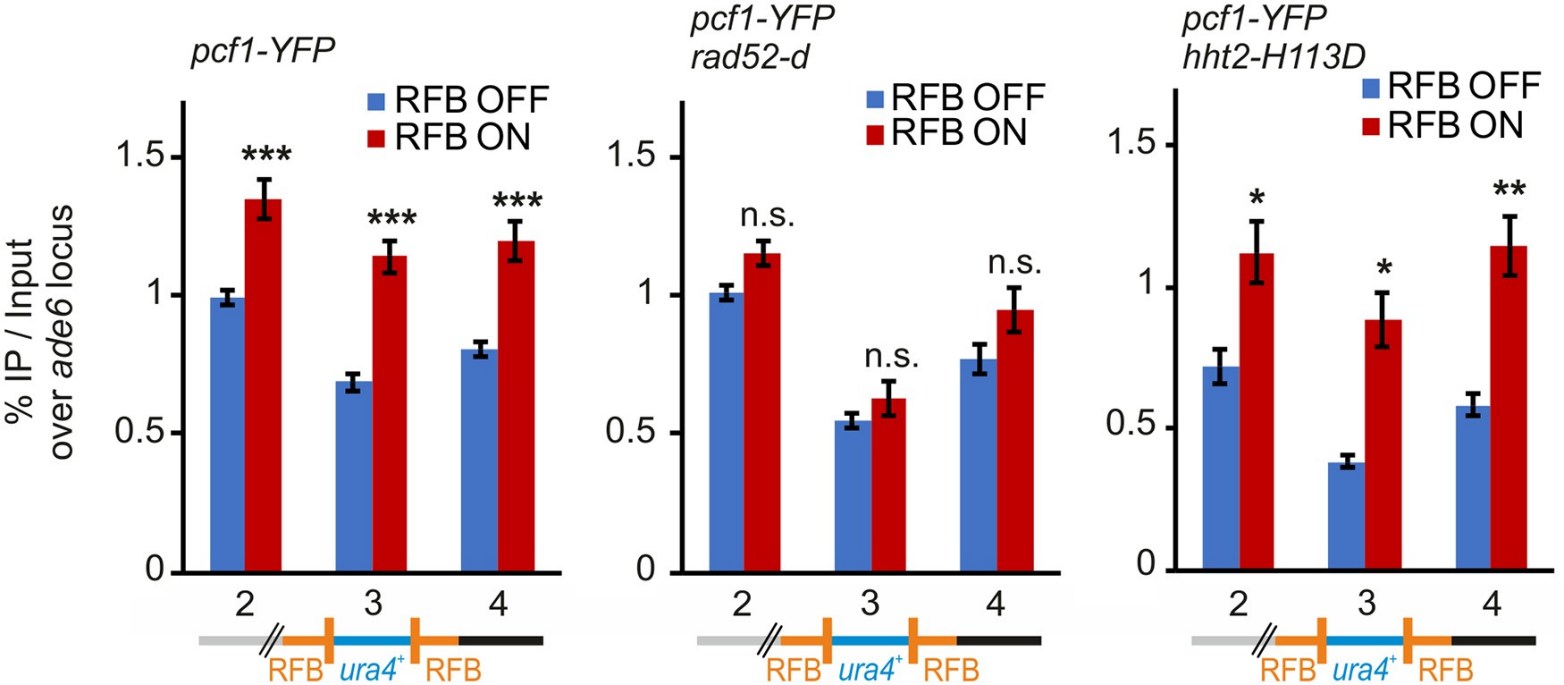
CAF-1 association to HR-mediated DNA synthesis requires Rad52. Pcf1-YFP binding to the *t>ura4<ori* locus in RFB OFF and ON conditions (left panel: *wt strain*, middle panel: *rad52-d* strain, right panel: *hht2-H113D* strain). Schematic at the bottom depicts the primers location within the *t>ura4<ori* locus: primer pair 2 is located 200 bp away from the telomere-proximal RFB; primer pair 3 is within the *ura4* gene; primer pair 4 is located 110 bp away from the centromere-proximal RFB. Values are means of at least 4 independent biological replicates ±SEM. Statistical analysis was performed using student t-test: * p<0.05, ** p<0.005, *** p<0.0005 compared to RFB OFF condition.

Our data indicate that CAF-1 and histone deposition promote RDR by stabilizing D-loop intermediates. Since DNA synthesis is primed from the 3’end of the invading strand within the D-loop, one could expect CAF-1 to associate with such sites of DNA synthesis *in vivo*. D-loops are transient DNA structures that form into the genome upon DNA damage. Therefore, we took advantage of the RDR assay in which the replication of the *ura4* gene is dependent on HR to ask if CAF-1 associates with this site of HR-dependent DNA synthesis. Pcf1-YFP was found enriched at the two RFB sites in ON condition, compared to OFF condition (Fig 5, primer pairs 2 and 4, left panel). Remarkably, the binding of Pcf1-YFP to *ura4* was stimulated upon activation of the RFB (Fig 5, primer pair 3, left panel). To test if such binding to RFBs depends on the formation of recombination intermediates, we repeated these analyses in *rad52-d* cells in which JMs do not occur [27]. Pcf1 binding to *ura4* and two RFB sites was no longer stimulated by the activation of the RFB in the absence of Rad52 (Fig 5, middle panel). We concluded that CAF-1 recruitment to HR-mediated DNA synthesis requires the formation of JMs. We asked if inhibiting (H3-H4)_2_ tetramer formation prevents CAF-1 binding to sites of HR-mediated DNA synthesis. Upon activation of the RFB, Pcf1-YFP was found enriched at the two RFBs and at *ura4* in cells expressing H3-H113D, showing that RDR defect in *hht2-H113D* strain is not a consequence of CAF-1 inability to bind the site of HR-mediated DNA synthesis. Altogether, our data demonstrate that CAF-1 associates to sites of HR-dependent DNA synthesis, downstream of Rad52 and JMs formation.

### RDR-coupled histone deposition promotes cell resistance to camptothecin in the absence of Rqh1

**Fig 6:**
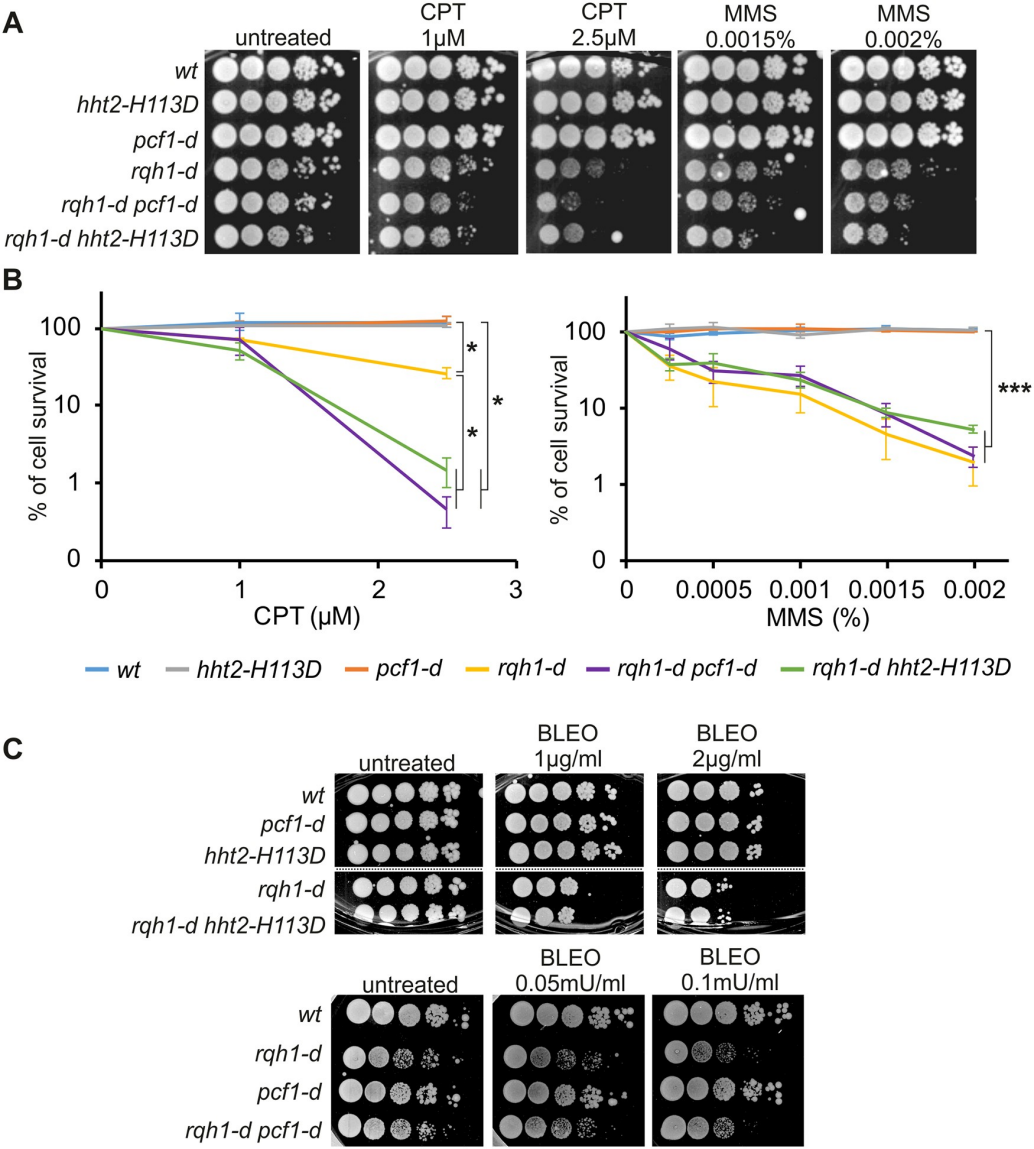
Histone deposition ensures cell survival to camptothecin in the absence of Rqh1. **(A)** Ten-fold serial dilution of indicated strains in indicated conditions of CPT and MMS. **(B)** Cell survival curves of indicated strains in indicated conditions. Values are means of at least 3 independent biological replicate ±SEM. Statistical analysis was performed using student t-test: * p<0.05, *** p<0.0005. **(C)** Ten-fold serial dilution of indicated strains in indicated conditions of bleomycin (BLEO).

We have addressed the role of fork-associated histone deposition to the cell response to replication stress. The H3-H113D mutation or the *pcf1* deletion decreased cell survival of *rqh1* deleted cells upon camptothecin (CPT, a topoisomerase I inhibitor) treatment, and resulted in a moderate synthetic growth defect upon MMS treatment (Fig 6A and 6B). This was not a consequence of a loss of Rqh1-Pcf1 interaction in cells expressing H3-H113D as Rqh1-MYC associated with Pcf1-YFP in *hht2-H113D* mutated cells (S4D Fig). Also, DNA damage did not stimulate chromatin-bound H3-H113D-HA, suggesting that unstable tetramers are unlikely to be assembled during repair synthesis (Fig 2F). Interestingly, such synthetic interactions were not observed in response to bleomycin (Fig 6C), a DSB-inducing agent. These data reveal that in the absence of Rqh1 activity, cell resistance to topoisomerase I inhibitor relies on CAF-1-mediated histone deposition.

### (H3-H4)_2_ tetramer formation favors deletion type recombinant in the absence of Rqh1

**Fig 7:**
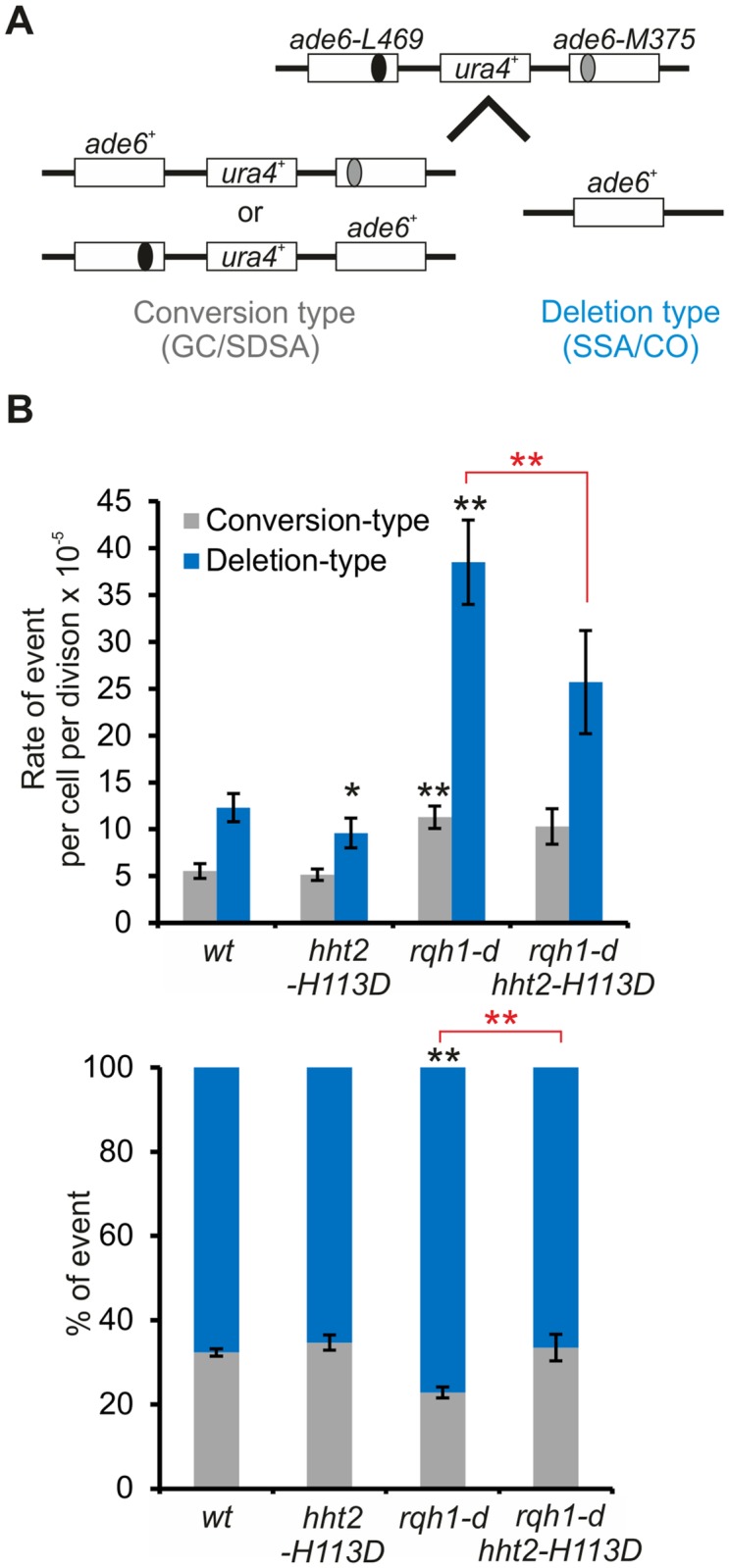
Histone deposition favors deletion-type events. **(A)** Schematic representation of HR substrate and recombination outcomes. **(B)** Top panel: rate of conversion and deletion type in indicated strains. Values are median rate calculated from 23 to 26 independent cultures ± 95% confidence interval (CI). Bottom panel: ratio of deletion and conversion type in indicated strains. Error bars indicate SEM. Statistical analysis was performed using Mann & Whitney U test. Black and red stars indicate statistics compared to *wt* and compared to *rqh1-d*, respectively. * p<0.01, ** p<0.001.

We asked if the role of fork-associated histone deposition in promoting HR event might also occur independently of Rqh1. The lack of Rqh1 results in a spontaneous hyper-recombination phenotype caused by its multiple roles in processing HR intermediates [50]. We asked if histone deposition contributes to the anti-recombinase activity of Rqh1 by monitoring spontaneous HR events using an assay for intra-allelic recombination between direct *ade6* repeats (Fig 7A) [51]. Gene conversion (GC) and synthesis dependent strand annealing (SDSA) result in conversion type, whereas crossover (CO) and single strand annealing (SSA, a DNA repair pathway independent of Rad51-mediated strand exchange) give rise to deletion type. In *rqh1-d* cells, conversion and deletion rate were increased by 2- and 3-fold, respectively, which is consistent with known Rqh1 anti-recombinase activities (Fig 7B) [50]. The *hht2-H113D* mutation had minor impact on HR outcomes but when combined with *rqh1-d*, it decreased by 35% the rate of deletion type without affecting the rate of conversion type (*hht2-H113D* and *rqh1-d hht2-H113D* compared to *wt* and *rqh1-d* respectively). These data establish that a significant part of the deletion events occurring in the absence of Rqh1 are caused by (H3-H4)_2_ tetramer formation. Thus, histone deposition also promotes HR events by other mechanisms than counteracting Rqh1 activity. These data are consistent with antagonistic activities of Rqh1 and histone deposition in controlling HR outcomes: Rqh1 promotes D-loop disassembly to favor a conservative pathway whereas histone deposition promotes D-loop stability to favor deletion events such as CO.

## Discussion

**Fig 8:**
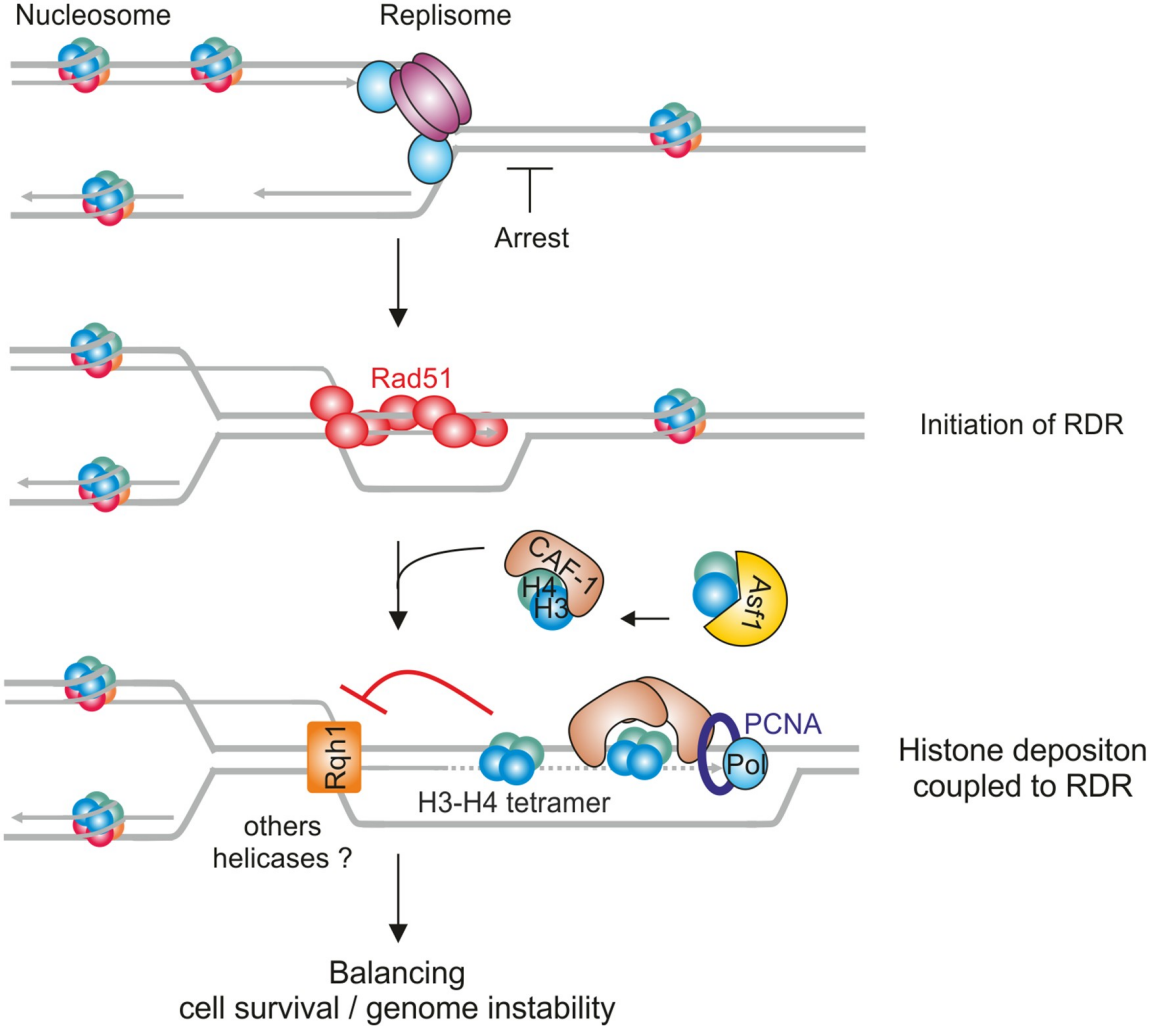
Model of histone deposition coupled to HR-mediated DNA synthesis at arrested forks. Model of histone deposition coupled to recombination-dependent replication. Upon fork arrest, HR factors promote D-loop formation which primes the restarted DNA synthesis and histone deposition. Histone deposition coupled to RDR allows JMs to be protected from disassembly by DNA helicases such as Rqh1. This fork-associated histone deposition pathway to stabilize recombination intermediates contributes to replication recovery and balances genome stability at site of replication stress.

Fork-obstacles open up the risk of genome instability. HR ensures the timely completion of genome duplication by restarting dysfunctional forks, but this comes at the expense of TS events that can generate genome rearrangements. Here, we established that HR-mediated DNA synthesis is coupled to histone deposition, mediated by CAF-1 and Asf1, and that this process is necessary to promote RDR (Fig 8). We established that inhibiting (H3-H4)_2_ tetramer formation impacts RDR and D-loop resolution, in a manner dependent on CAF-1 function. Our data support a mechanism during which RDR is coupled to CAF-1-mediated histone deposition to stabilize D-loop intermediates to promote RDR. We reveal a novel interplay between chromatin restoration and DNA repair factors; a crosstalk necessary to cell survival to replication stress. Our finding highlight that chromatin restoration is an integral part of the RDR process. We propose that histone deposition plays an active role in RDR to avoid discontinuity in chromatin assembly upon replication stress, a benefit counterbalanced by stabilizing at-risk joint-molecules for genome stability.

A complex network of histone chaperones handles histone dynamics at replication forks to promote chromatin assembly. We establish that only a subset of this network is pivotal to promote RDR. Despite recent advances in understating the mechanism of CAF-1-mediated histone deposition *in vitro*, it remains challenging to define point mutations to generate mutated forms of CAF-1 unable to interact with H3-H4 *in vivo* [12]. To overcome this, we took advantage of the H3-H113D mutated form, reported to inhibit CAF-1-mediated histone deposition *in vitro* [36]. We show that H3-H113D precludes (H3-H4)_2_ tetramer formation, is poorly incorporated into nucleosomes assembled onto DNA and alters the organization of the newly replicated chromatin. Remarkably, the H3-H113D mutation mimics the absence of CAF-1 in impairing RDR. We cannot exclude that H3-H113D has potential CAF-1-independent effects, but we have observed no additive effect between the lack of CAF-1 and H3-H113D indicating that the inhibition of replication-coupled histone deposition is responsible for the defect in RDR. RDR requires Asf1, the 3 CAF-1 subunits and the ability of CAF-1 to interact with PCNA [34], supporting the view that Asf1 and CAF-1 promote RDR by coupling histone deposition to the step of DNA synthesis. Consistent with this, CAF-1 binds to site of HR-mediated DNA synthesis in a Rad52-dependent manner, indicating that the early steps of RDR must be engaged for the subsequent recruitment of CAF-1. The binding of Rad52 to arrested forks is not affected when inhibiting histone deposition or in the absence of CAF-1 [34], consistent with CAF-1-mediated histone deposition acting downstream of Rad52.

We proposed that CAF-1-mediated histone deposition acts as a chromatin restoration pathway during the DNA synthesis step of RDR. In this scenario, histone deposition would occur onto the DNA duplex of the extended D-loop (Fig 8). We cannot exclude that histone deposition occurs onto the displaced strand of the D-loop, as it has been proposed that nucleosome can be deposited onto ssDNA [52]. RDR requires two histone chaperones that mediate chromatin assembly in a DNA synthesis manner and requires the ability of CAF-1 to interact with PCNA. Therefore, we favor the first hypothesis in which histone deposition is coupled to DNA synthesis during D-loop extension. Extensive works have address the role of chromatin factors in regulating DNA lesions accessibility to DNA repair machineries but how chromatin restoration is coupled to the DNA repair event is poorly understood [3]. Our data put forward a crosstalk between the DNA repair machinery and the step of chromatin restoration that plays an active role in promoting RDR.

Histone deposition coupled to RDR impacts D-loop resolution. One mechanism involves nucleosome assembly onto D-loop intermediates to counteract Rqh1 activity. Possibly, histone deposition onto extended D-loops creates a substrate less favorable to the Rqh1 activity. RecQ helicases act as motor proteins/helicases to migrate DNA junctions which may be prevented by assembled nucleosomes. It remains challenging to address whether histones are deposited onto D-loops as these structures are very transient. JMs visualization by 2DGE requires a step of enrichment in replication intermediates, technically incompatible with chromatin immuno-precipitation approaches to address histone binding to JMs. Nonetheless, we establish that inhibiting replication-coupled histone deposition favors D-loop disassembly. We provide insights into the nature of the chromatin required to protect D-loops. Asf1 acts as an histone chaperone to present H3-H4 to Rtt109 and generates the H3K56Ac mark and then transfer H3-H4 to CAF-1 [7]. We found H3K56Ac being dispensable to promote TS during the initiation of RDR. Possibly, Asf1 acts as a donor histone to CAF-1. However, H3-H113D binds to CAF-1 likely as a H3-H4 dimer but not to Asf1, indicating that H3-H4 can be handed off to CAF-1 independently of Asf1, a pathway that remains to be clarified.

Nucleosome deposition onto extended D-loop may impose topological constraints. D-loop disassembly is a topoisomerase-mediated mechanism [53]. Thus, topological constraints resulted from DNA wrapped around nucleosome may be easily relieved by topoisomerase 3. When seeking for additional chromatin factors required for RDR, we found Nap1 and Nap2, two histone H2A-H2B chaperones, to be dispensable to protect D-loop. This suggests that the deposition of (H3-H4)_2_ tetramer, but not the formation of a nucleosome, is sufficient by itself to counteract Rqh1 activity and to limit topological constraints.

Fork-associated histone deposition affects HR outcomes by mechanisms that are also independent of Rqh1 activity. During HR-mediated DSB repair, disassembly of D-loops extended by DNA polymerase ensures a non-crossover outcome [26]. We report that in the absence of Rqh1, a significant part of the deletion type events is favored by histone deposition. Chromatin assembly at D-loop intermediates may also counteract the activity of other helicases in disrupting JMs [26]. Interestingly, spontaneous rates of gene conversion were unaffected by inhibiting histone deposition. This suggests that DNA synthesis associated to GC and SDSA is too short in length to favor histone deposition. Nonetheless, our data reveal that the antagonistic activities of RDR-coupled histone deposition and Rqh1 in D-loop resolution are pivotal to balance genome stability at arrested forks and to promote cell resistance upon replication stress.

During unchallenged replication, the concerted action of multiple histone chaperones coordinates the assembly of chromatin behind the fork to achieve recycling of parental histones and deposition of newly synthetized histones [20]. RDR results in the progression of non-canonical forks in which both strands are synthetized by the DNA polymerase delta, which contrasts with the division of labor between DNA polymerase delta and epsilon at origin-born replication forks [54]. Such restarted forks are liable to replication errors such as multiple template switches, replication slippage and U-turn [32,33,55]. Despite these unusual features, our data suggest that restarted forks remain coupled to histone deposition and thus may help to ensure continuous chromatin assembly upon replication stress.

Fork obstacle and replication stress interfere with the inheritance of epigenetic marks [23,56,57]. On the one hand, the post-replicative repair of DNA lesions/structures, left un-replicated behind the fork, is uncoupled from chromatin assembly and recycling of parental histones. On the other hand, it was proposed that the bypass of DNA secondary structures, such as G quadruplexes, by PrimPol allows the repriming of DNA synthesis very closely to the fork obstacle, allowing for the maintenance of replication-coupled chromatin maturation [58]. The choice of the pathway employed to overcome fork obstacles may influence the maintenance of histone deposition coupled to restarted forks. Our data indicate that RDR, a main pathway to bypass fork obstacles, is coupled to histone deposition. In the view that replisomes are often interrupted by numerous obstacles, RDR-coupled histone deposition contributes to cell resistance to replication stress and may ensure continuity in assembling chromatin upon replication stress. Nonetheless, this comes at the expense of stabilizing JMs which can be detrimental to genome stability.

## Materials and methods

### Standards yeast genetics

Yeast strains used in this work are listed in S1 Table. Gene deletion and gene tagging were performed by classical and molecular genetics techniques [59]. Strains containing the *RTS1*-RFB were grown in supplemented EMM-glutamate media containing 60μM of thiamine. To induce the *RTS1*-RFB, cells were washed twice in water and grown in supplemented EMM-glutamate media containing thiamine (Rtf1 repressed, RFB OFF condition) or not (Rtf1 expressed, RFB ON condition) for 24 hours or 48 hours.

### Chromatin fraction assay

Chromatin fractionation was performed as described previously [60] with the following modifications: Cells were harvested, digested with 100μg of zymolyase 20T (Amsbio, 120491–1) to obtain spheroplasts, and resuspended in lysis buffer (50mM potassium acetate, 2mM MgCl2, 20mM HEPES pH7.9, 1% Triton X-100, 1mM PMSF, 60mM β-glycerophosphate, 0.2mM Na_3_VO_4_, 1μg/ml AEBSF and Complete Protease Inhibitor EDTA-Free Tablet (Roche, 04 693 159 001). After lysis, extracts were subsequently fractionated into soluble and pellet fractions by centrifugation. The insoluble chromatin-enriched pellet fraction was washed twice with the lysis buffer without 1% Triton X-100 and digested with 100Units of DNase I HC (ThermoScientific, EN0523) on ice for 15min followed by 15min at RT. The DNase I-digested chromatin-enriched fraction was centrifuged for 5 min at 16,000g. Supernatant was designated as the chromatin fraction. Samples corresponding to total soluble and chromatin fractions were migrated and transferred on nitrocellulose membrane. Proteins of interest were revealed by anti-HA (1/500^e^, SantaCruz, 12CA5), anti-PCNA (1/500^e^, SantaCruz, PC10), anti-Tubulin (1/4000^e^, Abcam, ab6160) and anti-H3 (1/2000^e^, Abcam, ab1791) antibodies.

### Analysis of replication intermediates by 2DGE

Replication Intermediates (RIs) were analyzed by 2DGE as previously described [61]. RIs were migrated in 0.35% agarose gel in 1X TBE for the first dimension. The second dimension was migrated in 0.9% agarose gel 1X TBE supplemented with EtBr. DNA was transferred onto a nylon membrane (Perkin Elmer, NEF988001PK) in 10X SSC. Membranes were incubated with a ^32^P radiolabeled *ura4* probe, and RIs were detected using phosphor-imager software (Typhoon-trio) and quantified with ImageQuantTL.

### Co-immunoprecipitation

5.10^8^ cells were harvested with 0.1% sodium azide, washed in cold water and resuspended in 400 μl of EB buffer (50mM HEPES High salt, 50mM KOAc pH7.5, 5mM EGTA, 1% Triton X-100, 1mM PMSF, and Complete Protease Inhibitor EDTA-Free Tablet (Roche, 04 693 159 001). Cell lysis was performed with a Precellys24 homogenizer (Bertin instruments). The lysate was treated with 250mU/μl of benzonase (Novagen, NOVG 70664–3) for 30min. After centrifugation, the supernatant was recovered and an aliquot of 50 μl was saved as the INPUT control. To 300μl of lysate, 2μl of anti-GFP antibody (Life Technologies, A11122) were added and incubated for 1.5 hours at 4°C on a wheel. Then, 20μl of Dynabeads protein-G (Life Technologies, 10004D) prewashed in PBS were added and then incubated at 4°C overnight. Alternatively, lysates were incubated overnight with 20μl of anti-MYC (Life Technologies, 88842) or anti-HA (Life Technologies, 88836) antibody coupled to magnetic beads. Proteins of interest were detected using anti-HA high affinity (1/500e, Roche, clone 3F10), anti-GFP (1/1000e Roche, 11 814 460 001), anti-MYC (1/300e, SantaCruz, A-14), anti-HA (1/500e, SantaCruz, 12CA5), anti-PCNA (1/500e, SantaCruz, PC10), and anti-H3 (1/2000e, Abcam, ab1791) antibodies. Co-immunoprecipitation experiments were quantified with ImageLab software (BioRad). Intensities of bands were normalized with the background.

### Chromatin immunoprecipitation of Pcf1-YFP

ChIP experiments were performed as previously reported [62]. 10^9^ cells were crosslinked with fresh 1% formaldehyde (Sigma, F-8775) for 15 minutes. Cells were lysed using Precellys24 homogenizer (Bertin instruments) in lysis buffer (50mM Hepes-KOH pH7.5, 140mM NaCl, 1mM EDTA, 1% Triton X-100, 0.1% sodium deoxycholate, 1mM phenylmethylsulfonyl fluoride, and Protease Inhibitor Cocktail (Sigma P8215)). The crude cell lysate was sonicated (using a Diagenod Bioruptor at high setting for 15 cycles: 30 seconds ON + 30 seconds OFF) and clarified by centrifugation for 15min at 16,000g. Prior to immunoprecipitation, 1/100 volume of the cell lysate was saved for an input control. Immunoprecipitations were performed with 2μl of anti-GFP antibody (Life Technologies, A11122) for 2 hours. After 30min incubation with 20μl magnetic beads (Life Technologies, 10004D), immunoprecipitates were successively washed with 2x1ml lysis buffer, 2x1ml lysis buffer/500mM NaCl, 2x1ml wash buffer (10mM Tris-HCl pH8, 0.25M LiCl, 0.5% NP-40, 0.5% sodium deoxycholate, 1mM EDTA) and 1ml TE buffer (10mM Tris-HCl, 1mM EDTA pH8). Crosslinks were reversed by incubating the samples at 65°C overnight. Samples were then treated with 0.5mg/ml Proteinase K (Euromedex, EU0090) and DNA was purified using Qiagen PCR purification kit and eluted in 100μl of water. The relative amount of DNA was quantified by qPCR (primers are listed in S2 Table). Pcf1-YFP enrichment was normalized to an internal control locus (*ade6*).

### Chromatin immunoprecipitation of Rad52

Rad52 ChIP experiments were performed as previously reported [63]. 2.10^8^ cells were fixed with 1% formaldehyde (Sigma, F-8775), lysed in 500μl breaking buffer (0.1M Tris-HCl pH8, 20% glycerol, 1mM phenylmethylsulfonyl fluoride) using a Precellys24 homogenizer (Bertin instruments) and sonicated with a Diagenod Bioruptor at high setting for 15 cycles: 30 seconds ON + 30 seconds OFF. Immunoprecipitations (IP) were carried out overnight at 4°C in ChIP buffer (50mM Hepes-KOH pH7.5, 140mM NaCl, 1mM EDTA, 1% Triton X-100, 0.1% sodium deoxycholate, 0.1% SDS, 1mM phenylmethylsulfonyl fluoride) using 1μl of anti-Rad52 antibody (Abcam, Ab63800). Protein G magnetic beads (Life Technologies, 10004D) were then added to the samples and incubated for 4h at 4°C. IPs were successively washed with 1ml ChIP buffer, 2x1ml ChIP buffer/500mM NaCl, 2x1ml wash buffer (10mM Tris-HCl pH8, 0.25M LiCl, 0.5% NP-40, 0.5% sodium deoxycholate, 1mM EDTA, 1mM phenylmethylsulfonyl fluoride) and 1ml TE buffer (10mM Tris-HCl, 1mM EDTA pH8). Crosslinks were reversed for both IP and Input samples by incubation at 65 °C overnight. Samples were then treated with 0.5mg/ml Proteinase K (Euromedex, EU0090) and DNA was purified by phenol/chloroform extraction. Purified DNA were analyzed by real-time quantitative PCR (CFX96 Real-Time PCR Detection System and iQ SYBR Green Supermix, BioRad) using those cycling conditions: 95°C for 3 min; 40 cycles of 95°C for 15 seconds, 60°C for 1 min. Primers used in this study are listed in S2 Table.

### Pulse-Field Gel Electrophoresis

PFGE were performed as previously described [27]. Membranes were then incubated with a ^32^P radiolabeled *rng3* probe. Quantification of acentric chromosomes visualized by PFGE was performed as previously described [34].

### Microccocal digestion and BrdU incorporation

Microccocal digestions were performed as previously described [64]. After crosslink with 1% formaldehyde, 1.10^9^ Cells were spheroplasted in 2ml of CES buffer (50mM Citric acid/50mM Na2HPO_4_ pH 5.6, 40mM EDTA pH8, 1.2M Sorbitol, 20mM β-mercaptoethanol) containing 1 mg/ml Zymolyase 100T (Amsbio, 120493–1) for 20min at 30°C. Spheroplasts were washed twice with 1ml of iced cold 1.2M Sorbitol buffer. Cells were resuspended in 1ml of NP-S buffer (1.2M Sorbitol; 10mM CaCl_2_, 100mM NaCl, 1mM EDTA pH8, 14mM β-mercaptoethanol, 50mM Tris-HCl pH8, 0.075% NP-40, 5mM spermidine, 0.1mM PMSF, Complete Protease Inhibitor EDTA-Free Tablet (Roche, 04 693 159 001)) containing the indicated units of Micrococcal Nuclease (Worthington Biochemical, LS004798) for 10 min at 37°C. Reactions were stopped by addition of 50mM EDTA pH8 and SDS 0.2%. Crosslinks were reversed overnight at 65°C in the presence of 20μg of RNAseA (Sigma, R5503) and 0.2mg/ml Proteinase K (Euromedex, EU0090). DNA was purified by phenol/chloroform extraction and ethanol precipitation. Purified DNA was resolved on 1.5% agarose gel (1X TBE).

For BrdU incorporation, we used cells expressing *Drosophila melanogaster* deoxyribonucleoside kinase (DmdNK) under the control of the fission yeast a*dh* promoter, together with the human equilibrative nucleoside transporter (hENT1) (*adh-dmdNK-adh-hENT1*) [44]. Cells were arrested 4 hours with 20mM HU (Sigma, H8627) and released in fresh media containing BrdU (Sigma, B5002) for 20min. After MNase digestion, DNA was analyzed by Southern-blot using anti-BrdU antibody (1/4000^e^, Abcam, ab12219).

### Spontaneous recombination assay

Spontaneous recombination rate was assayed using strains containing a direct repeat of two nonfunctional *ade6* alleles flanking a functional *ura4* gene [51]. Strains were kept on low adenine EMM plates lacking uracil to prevent selection for Ade^+^ and Ura^-^ recombinants. Dark pink colonies were streaked on supplemented YE plates and 23 to 26 independent single colonies for each strain were used to calculate Ade+ recombinant rate. Appropriate dilutions were plated on supplemented YE plates (to determine cell survival), EMM plates lacking adenine (to score spontaneous recombination rate, Ade+ recombinants) and EMM plates lacking adenine and uracil (to score gene conversion rate, Ade+ Ura+ recombinants). Colonies were counted after 5–7 days of incubation at 30°C. The rates of Ade+ and Ade+ Ura+ recombinant were calculated as described in [65].

### Analyzed X-ray structures of nucleosome

There is no experimental model of nucleosome containing histones from *S*. *pombe*. Among the numerous available structures of nucleosome, only three of them include yeast histones, all from *S*. *cerevisiae* (PDB codes 1ID3, 4JJN and 4KUD), with resolutions of ~3 Å. Indeed, the highest resolution was obtained for a nucleosome containing histones from *Xenopus laevis* (PDB code 1KX5, resolution of 1.9 Å). Since the histone sequences are extremely conserved, *S*. *cerevisiae* and *Xenopus laevis* H3 histones share 92% of residues with H3 of *S*. *pombe*. More specifically, the region surrounding H113 is well preserved across *S*. *pombe*, *S*. *cerevisiae* and *Xenopus laevis*, with a very good score being observed for the couple *S*. *pombe / Xenopus laevis* (S2A Fig). Given the reasonable sequence agreement, 1ID3, 4JJN, 4KUD and 1KX5 were analyzed using PDBsum to describe the interface between two H3-H4 dimers [66].

### Data availability

Numerical data used for graphs are provided in the supplementary information S1 Data.

## Supporting information

S1 Fig Chromatin factors involved in RDR (related to Fig 1). **(A)** Ten-fold serial dilution of indicated strains in indicated conditions. **(B)** Chromosome analysis in indicated strains and conditions by PFGE and Southern-blot using a radiolabeled *rng3* probe. **(C)** Quantification of acentric level normalized to chromosome III level. Values are means of at least 3 independent biological replicates ±SEM. Statistical analysis was performed using Student’s t-test: * p<0.05, *** p<0.0005, compared to *wt*.
(TIF)

S2 Fig Impact of H3-H113D on interactions with *wild type* H3 (related to Fig 2). **(A)** Amino acid sequences of the region containing H113 and its interacting partners in the H3:H3’ nucleosomal interface. This table gives the amino acid sequences of the relevant part of H3 in *Schizosaccharomyces pombe* and the considered X-ray structures. The X-ray structures are referenced by their PDB codes. 1KX5 contains histones from *Xenopus laevis*; 1ID3, 4JJN and 4KUD include histones from *Saccharomyces cerevisiae*. The residues in red differ from those of *Schizosaccharomyces pombe*. The residues on yellow background form a network of contact with H113 (green background) in the H3:H3’ interface. **(B)** Schematic representation of the contact network involving H113 in the H3:H3’ interface. H3’-H113 interacts with 6 residues of H3. Two hydrogen bonds (red arrows) are reinforced by Van der Waals contacts (grey arrows). Identical, symmetric interaction pattern is observed with H3-H113 and C110, H3’-D123, A114, R116, K122 and L126. The interface analysis was carried out with PDBsum (44). **(C)** Top panels: effect of the H113D mutation on histone H3-HA level transcribed from *hht1*, *hht2* or *hht3*. Tubulin was used as loading control. Bottom panels: Quantification of top panels: the level of H3-H113D-HA and H3-HA were normalized to tubulin. Individual values are plotted ± the range.
(TIF)

S3 Fig BrdU incorporation during and after HU treatment (related to Fig 3). Logarithmic growing cells from SL1077 strain were arrested in early S-phase with HU treatment. A pulse of 20min BrdU (400μM) incorporation was done after 4 hours of HU block or after releasing cells in a fresh media. Top panel: BrdU-incorporated genomic DNA was digested with increasing amount of MNase and migrated on ethidium bromide-containing agarose gel. Bottom panel: After transfer onto nitrocellulose membrane, incorporated BrdU was revealed using anti-BrdU antibody.
(TIF)

S4 Fig Impact of H3-H113D on interactions with histone chaperones (related to Fig 3). **(A)** Spore viability analysis of indicated genotypes. **(B)** Association of Asf1-MYC with histone H3 (H3, H3-HA and H3-H113D-HA). **(C)** Left panel: association of Pcf1-YFP with Pcf2-MYC in indicated strains. Right panel: quantification expressed in arbitrary unit (a.u.). Individual values are plotted ± the range. **(D)** Left panel: association of Pcf1-YFP with Rqh1-MYC and PCNA in indicated strains. Right panels: quantifications. Individual values are plotted ± the range. **(E)** Left panel: association of Pcf1-YFP with histone H3 and H4 in indicated strains. Right panels: quantifications. Individual values are plotted ± the range.
(TIF)

S5 Fig H3K56Ac is dispensable to promote TS during the initiation of RDR (related to Fig 4). **(A)** Level of H3K56Ac, H3-HA and H3-H113D in indicated strains. H3 and tubulin were used as loading control. Each panel corresponds to replicate loading on the same gel. **(B)** Level of H3 and H3-K56Ac in indicated strains. PCNA was used as a loading control. The two top and the two bottom panels correspond to the same samples loaded on two distinct gels. Each membrane was blotted with two different antibodies. **(C)** Chromosome analysis in indicated strains and conditions by PFGE and Southern-blot using a radiolabeled *rng3* probe. **(D)** Quantification of acentric level normalized to chromosome III level. Values are means of at least 4 independent biological replicates ±SEM. Statistical analysis was performed using Student t-test: *** p<0.0005 compared to *wt*.
(TIF)

S1 Table Strains used in this study (related to all figures). (DOCX)

S2 Table List of primers used in this study. (DOCX)

S1 Data Excel file containing numerical raw data. (XLSX)
