# Supplementary figures and images for: Histone deposition promotes recombination-dependent replication at arrested forks
Source: PLoS Genet. 2019 Oct 4;15(10):e1008441. doi: 10.1371/journal.pgen.1008441 (PMC6795475; doi:10.1371/journal.pgen.1008441)

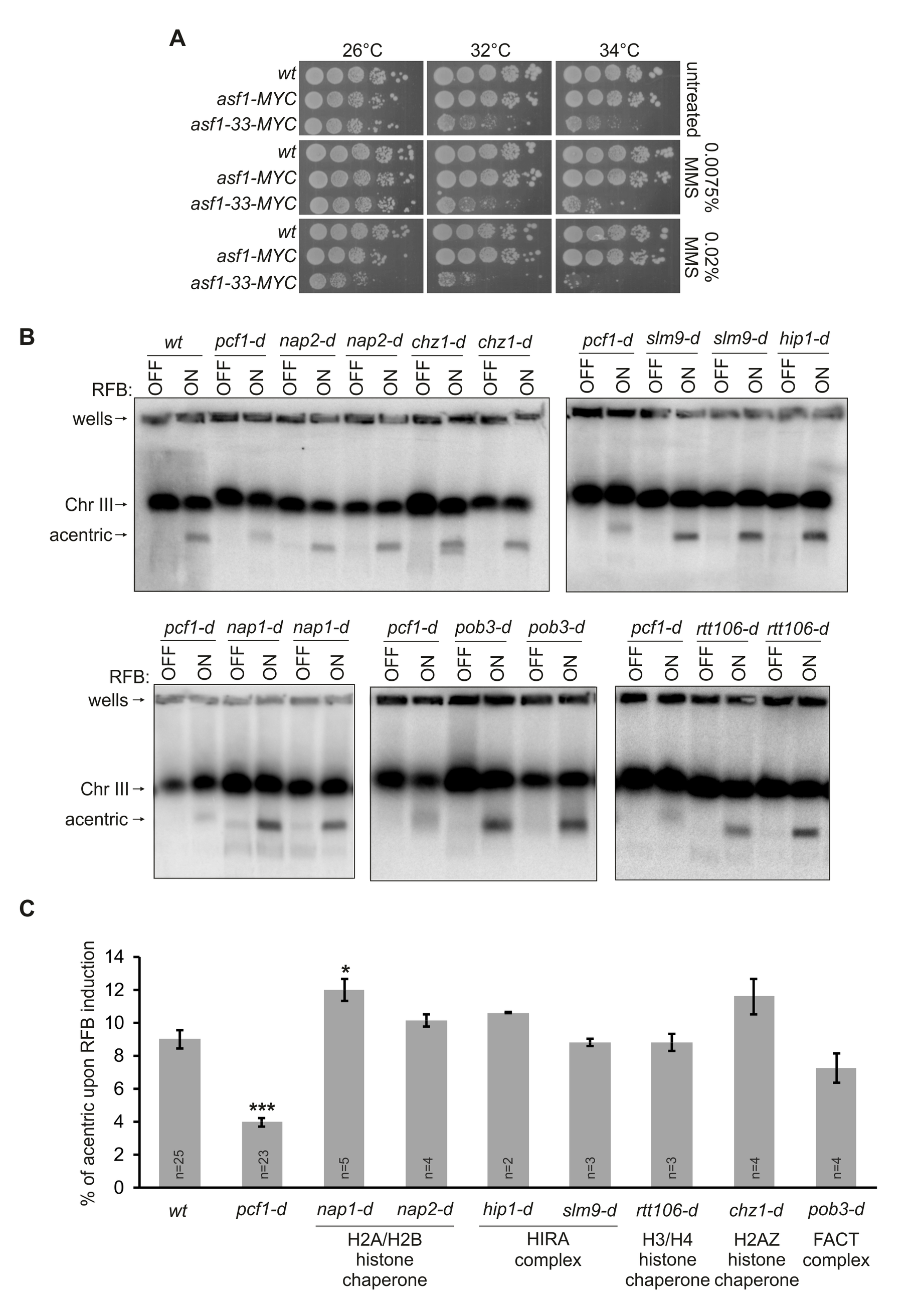

Supplement: S1 Fig — (A) Ten-fold serial dilution of indicated strains in indicated conditions. (B) Chromosome analysis in indicated strains and conditions by PFGE and Southern-blot using a radiolabeled rng3 probe. (C) Quantification of acentric level normalized to chromosome III level. Values are means of at least 3 independent biological replicates ±SEM. Statistical analysis was performed using Student’s t-test: * p<0.05, *** p<0.0005, compared to wt. (TIF) [file pgen.1008441.s001.tif]

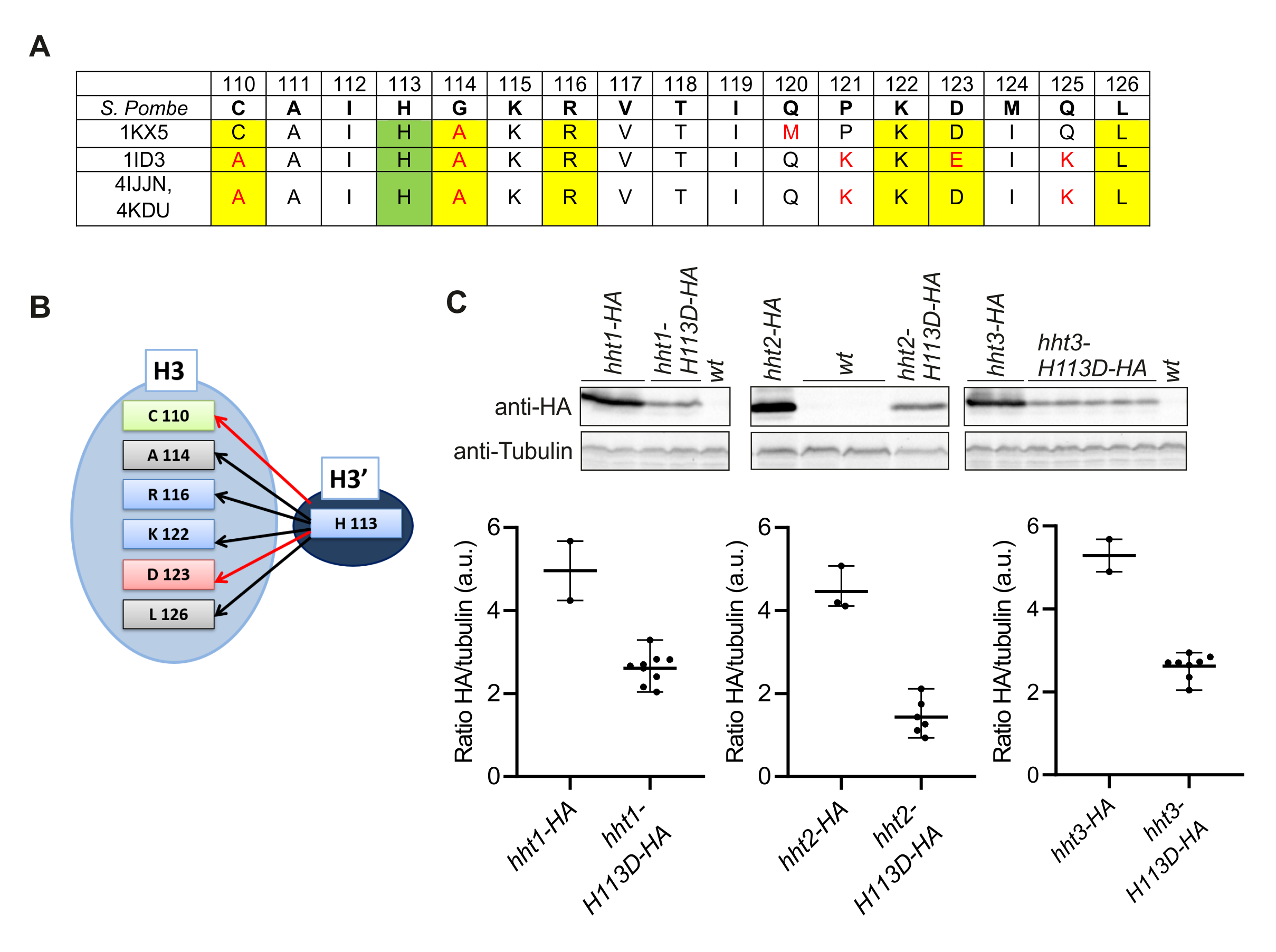

Supplement: S2 Fig — (A) Amino acid sequences of the region containing H113 and its interacting partners in the H3:H3’ nucleosomal interface. This table gives the amino acid sequences of the relevant part of H3 in Schizosaccharomyces pombe and the considered X-ray structures. The X-ray structures are referenced by their PDB codes. 1KX5 contains histones from Xenopus laevis; 1ID3, 4JJN and 4KUD include histones from Saccharomyces cerevisiae. The residues in red differ from those of Schizosaccharomyces pombe. The residues on yellow background form a network of contact with H113 (green background) in the H3:H3’ interface. (B) Schematic representation of the contact network involving H113 in the H3:H3’ interface. H3’-H113 interacts with 6 residues of H3. Two hydrogen bonds (red arrows) are reinforced by Van der Waals contacts (grey arrows). Identical, symmetric interaction pattern is observed with H3-H113 and C110, H3’-D123, A114, R116, K122 and L126. The interface analysis was carried out with PDBsum (44). (C) Top panels: effect of the H113D mutation on histone H3-HA level transcribed from hht1, hht2 or hht3. Tubulin was used as loading control. Bottom panels: Quantification of top panels: the level of H3-H113D-HA and H3-HA were normalized to tubulin. Individual values are plotted ± the range. (TIF) [file pgen.1008441.s002.tif]

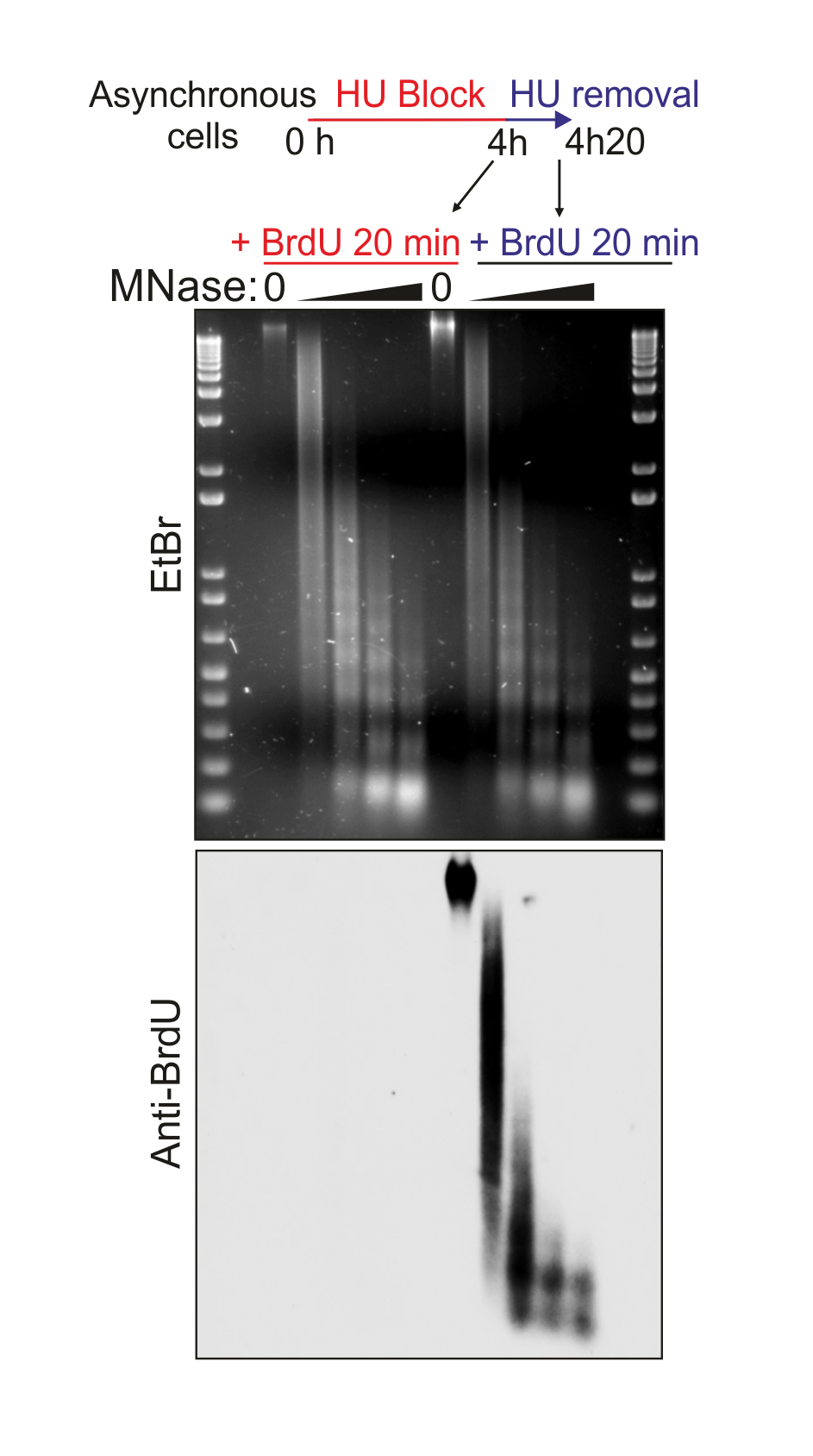

Supplement: S3 Fig — Logarithmic growing cells from SL1077 strain were arrested in early S-phase with HU treatment. A pulse of 20min BrdU (400μM) incorporation was done after 4 hours of HU block or after releasing cells in a fresh media. Top panel: BrdU-incorporated genomic DNA was digested with increasing amount of MNase and migrated on ethidium bromide-containing agarose gel. Bottom panel: After transfer onto nitrocellulose membrane, incorporated BrdU was revealed using anti-BrdU antibody. (TIF) [file pgen.1008441.s003.tif]

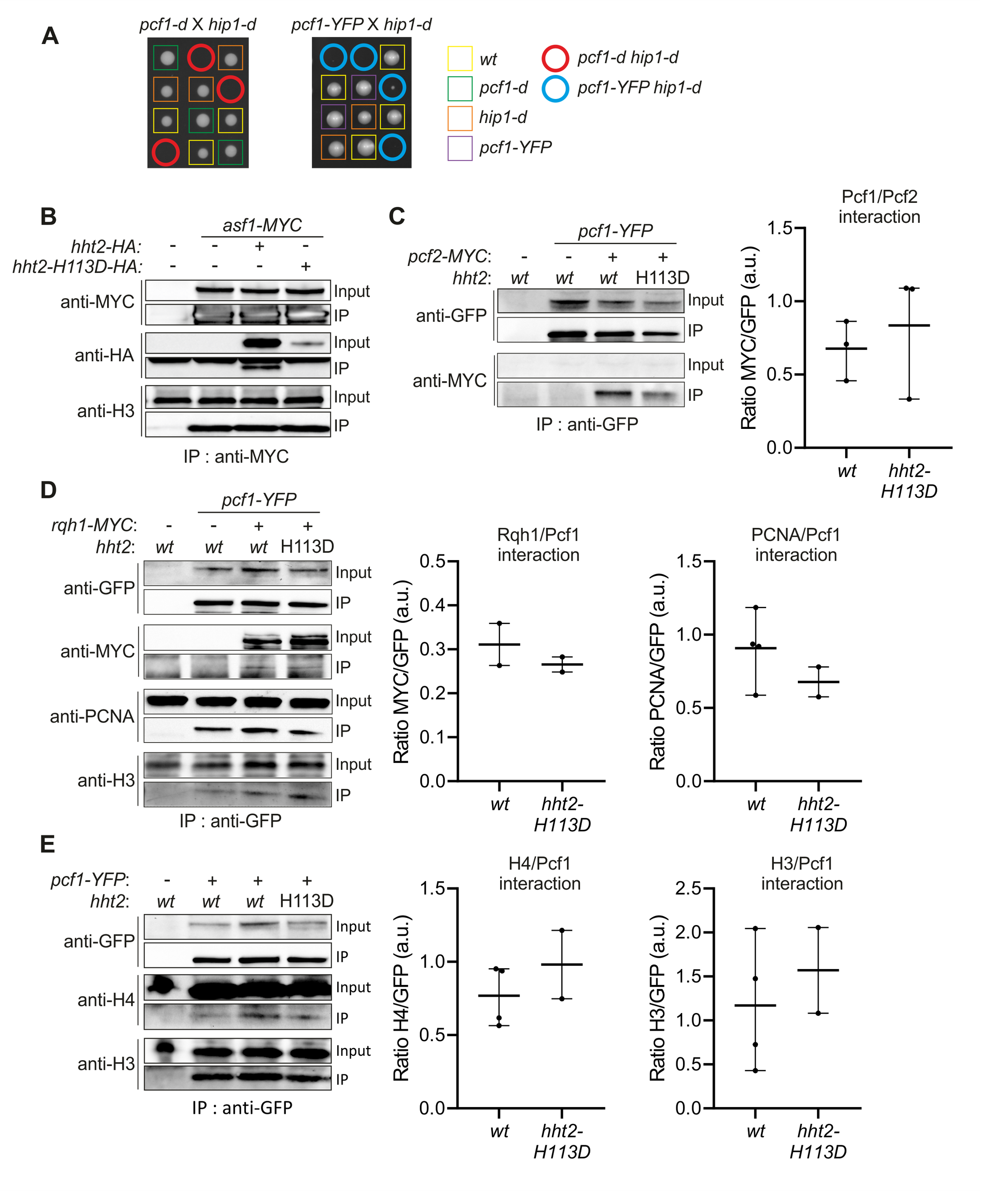

Supplement: S4 Fig — (A) Spore viability analysis of indicated genotypes. (B) Association of Asf1-MYC with histone H3 (H3, H3-HA and H3-H113D-HA). (C) Left panel: association of Pcf1-YFP with Pcf2-MYC in indicated strains. Right panel: quantification expressed in arbitrary unit (a.u.). Individual values are plotted ± the range. (D) Left panel: association of Pcf1-YFP with Rqh1-MYC and PCNA in indicated strains. Right panels: quantifications. Individual values are plotted ± the range. (E) Left panel: association of Pcf1-YFP with histone H3 and H4 in indicated strains. Right panels: quantifications. Individual values are plotted ± the range. (TIF) [file pgen.1008441.s004.tif]

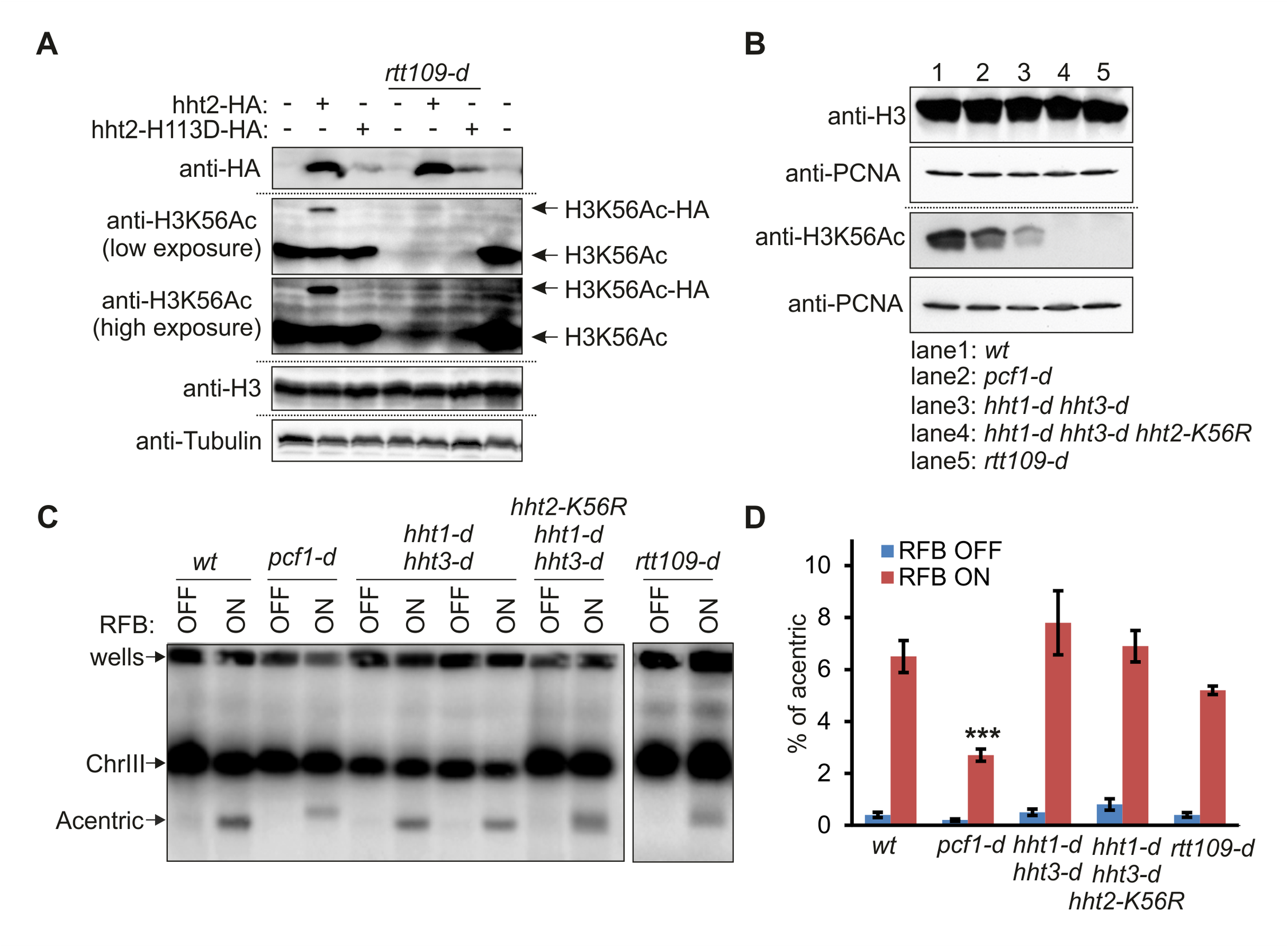

Supplement: S5 Fig — (A) Level of H3K56Ac, H3-HA and H3-H113D in indicated strains. H3 and tubulin were used as loading control. Each panel corresponds to replicate loading on the same gel. (B) Level of H3 and H3-K56Ac in indicated strains. PCNA was used as a loading control. The two top and the two bottom panels correspond to the same samples loaded on two distinct gels. Each membrane was blotted with two different antibodies. (C) Chromosome analysis in indicated strains and conditions by PFGE and Southern-blot using a radiolabeled rng3 probe. (D) Quantification of acentric level normalized to chromosome III level. Values are means of at least 4 independent biological replicates ±SEM. Statistical analysis was performed using Student t-test: *** p<0.0005 compared to wt. (TIF) [file pgen.1008441.s005.tif]
