# Supplementary material for: Histone deposition promotes recombination-dependent replication at arrested forks
Source: PLoS Genet. 2019 Oct 4;15(10):e1008441. doi: 10.1371/journal.pgen.1008441 (PMC6795475; doi:10.1371/journal.pgen.1008441)
Supplement: S1 Table — (DOCX) [file pgen.1008441.s006.docx]

**S1 Table**: **Strains used in this study (related to all figures)**

| Name |  | *Genotype* | | Reference | |  |
| --- | --- | --- | --- | --- | --- | --- |
| JH059 | *h- smto* | | *hht2-H113D:hphMX, ade6-704, leu1-32, ura4-D18* | | This study | |
| JH086 | *h- smto* | | *hht2-hhf2::kanMX6 , rtf1:nmt41:sup35, t>ura4^+^<ori, ade6-704, leu1-32* | | This study | |
| JH118 | *h- smto* | | *hht2-3HA:kanMX6, ade6-704, leu1-32, ura4-D18, his3-D1, arg3-D4* | | This study | |
| JH122 | *h- smto* | | *hht2-H113D-3HA:kanMX6, ade6-704, leu1-32, ura4-D18, his3-D1, arg3-D4* | | This study | |
| JH149 | *h- smto* | | *hht3-3HA-kanMX6, leu1-32, ura4-D18, ade6-704* | | This study | |
| JH151 | *h- smto* | | *hht3-H113D-3HA-kanMX6, leu1-32, ura4-D18, ade6-704* | | This study | |
| JH158 | *h- smto* | | *hht3-H113D-3HA-kanMX6, rtf1:nmt41:sup35, t>ura4^+^<ori, leu1-32, ade6-704* | | This study | |
| JH164 | *h+* | | *hht3-3HA-kanMX6, rtf1:nmt41:sup35, t>ura4^+^<ori, leu1-32, ade6-704* | | This study | |
| JH193 | *h- smto* | | *hht2-3HA:kanMX6, pcf1-YFP:kanMX6, ade6-704, leu1-32, ura4-D18, his3-D1, arg3-D4* | | This study | |
| JH197 | *h- smto* | | *hht2-H113D-3HA:kanMX6, pcf1-YFP:kanMX6, ade6-704, leu1-32, ura4-D18, his3-D1, arg3-D4* | | This study | |
| JH217 | *h+* | | *hht2-3HA:kanMX6, pcf1-YFP:kanMX6, asf1-MYC:kanMX6, leu1-32, ura4-D18, his3-D1, arg3-D4* | | This study | |
| JH218 | *h+* | | *hht2-H113D-3HA:kanMX6, pcf1-YFP:kanMX6, asf1-MYC:kanMX6, ade6-704, leu1-32, ura4-D18, his3-D1* | | This study | |
| JH242 | *h- smto* | | *hht2-H113D:hphMX, rqh1-MYC:kanMX6, pcf1-YFP:kanMX6, ade6-704, leu1-32, ura4-D18* | | This study | |
| JH245 | *h-smto* | | *hht2-H113D:hphMX, rqh1::kanMX6, ade6-704, leu1-32, ura4-D18* | | This study | |
| JH253 | *h+* | | *hht2-3HA:kanMX6, rtt109::kanMX6, ade6-704, leu1-32, ura4-D18* | | This study | |
| JH255 | *h+* | | *hht2-H113D-3HA:kanMX6, rtt109::kanMX6, ade6-704, leu1-32, ura4-D18, his3-D1, arg3-D4* | | This study | |
| JH274 | *h- smto* | | *hht2-H113D:hphMX6, pcf1-YFP:kanMX6, pcf2-4.5MYC:kanMX6, ade6-704, leu1-32, ura4-D18* | | This study | |
| JH300 | *h- smto* | | *rqh1::kanMX6, hht2-H113D:hphMX, rtf1:nmt41:sup35, t>ura4^+^<ori, ade6-704, leu1-32* | | This study | |
| JH302 | *h- smto* | | *nap2::kanMX6, rtf1:nmt41:sup35, t>ura4^+^<ori, ade6-704, leu1-32* | | This study | |
| JH304 | *h- smto* | | *chz1::kanMX6, rtf1:nmt41:sup35, t>ura4^+^<ori, ade6-704, leu1-32* | | This study | |
| JH305 |  | | *pob3::kanMX6, rtf1:nmt41:sup35, t>ura4^+^<ori, ade6-704, leu1-32* | | This study | |
| JH311 | *h- smto* | | *pcf1::kanMX6, ura4::adh::dmdNK-natMX-adh::hENT1, ura4-aim* | | This study | |
| JH312 |  | | *hht2-H113D:hphMX, ura4::adh::dmdNK-natMX-adh::hENT1, ura4-aim* | | This study | |
| JH318 | *h+* | | *hht2-H113D-HphMX, pcf1::kanMX6, ura4-aim, ura4::adh-dmNK-NAT-hENT1* | | This study | |
| JH336 | *h-smto* | | *rad52-GFP:kanMX6, pcf1::kanMX6, ade6-704, leu1-32, ura4-D18* | | This study | |
| JH341 | *h-smto* | | *rad52-GFP:kanMX6 hht2-H113D:hphMX6, ade6-704, leu1-32, ura4-D18* | | This study | |
| JH343 | *h+* | | *rad52-GFP-kanMX6, pcf1::kanMX6, hht2-H113D-hphMX6, bade6-704, leu1-32, ura4-D18* | | This study | |
| JH344 | *h+* | | *pcf1::kanMX6, hht2-H113D-hphMX6, rtf1:nmt41:sup35, t>ura4^+^<ori, ade6-704, leu1-32* | | This study | |
| JH359 |  | | *ade6-M375 int:puc8/ura4^+^/ade64-69, rqh1::kanMX6, ura4-D18* | | This study | |
| JH361 |  | | *ade6-M375 int:puc8/ura4+/ade64-69, hht2-H113D:hphMX6, ura4-D18* | | This study | |
| JH363 |  | | *ade6-M375 int:puc8/ura4+/ade64-69, rqh1::kanMX6, hht2-H113D:hphMX6, ura4-D18* | | This study | |
| JH403 | *h- smto* | | *pcf1-YFP:kanMX6 rad52::natMX6, rtf1::nmt41:sup35, t>ura4^+^<ori, ade6-704, leu1-32* | | This study | |
| JH408 |  | | *pcf1-YFP-kanMX6, hht2-H113D-HphMX, RTF1::nmt41:sup35, t>ura4^+^<ori, ade6-704, leu1-32* | | This study | |
| JH441 | *h+* | | *hht3-G9-5FLAG-HphMX, leu1-32, ura4-D18, ade6-704* | | This study | |
| JH443 | *h- smto* | | *hht3-G9-5FLAG-HphMX hht2-3HA-kanMX6, leu1-32, ura4-D18, ade6-704, his3-D1, arg3-D4* | | This study | |
| JH445 | *h- smto* | | *hht3-G9-5FLAG-HphMX hht2-H113D-3HA-kanMX6 leu1-32 ura4-D18 ade6-704 his3-D1 arg3-D4* | | This study | |
| SL75 | *h-* | | *ade6-704, leu1-32, ura4-D18,* | | Carr’s lab | |
| SL80 | *h+* | | *rqh1::kanMX6, ade6-704, leu1-32, ura4-D18* | | Pietrobon *et al.* 2014 | |
| SL228 | *h+* | | *rtf1:nmt41:sup35, t>ura4+<ori, ade6-704, leu1-32* | | Lambert *et al*. 2005 | |
| SL291 | *h+* | | *rad52-GFP:kanMX6, ade6-704, leu1-32, ura4-D18* | | Tsang *et al.* 2014 | |
| SL413 | *h+* | | *rad52::kanMX6, ade6-704, leu1-32, ura4-D18* | | Lambert *et al.* 2005 | |
| SL917 | *h+* | | *ade6-M375 int:puc8/ura4+/ade64-69* | | Hartsuiker et al. 2001 | |
| SL990 | *h-* | | *asf1-MYC:kanMX6, ade6-704, leu1-32, ura4-D18* | | Tanae *et al.* 2012 | |
| SL991 | *h+* | | *asf1-33-MYC:kanMX6, ade6-704, leu1-32, ura4-D18* | | Tanae *et al.* 2012 | |
| SL1077 | *h+* | | *ura4::adh::dmdNK-natMX-adh::hENT1, ura4-aim* | | Fleck *et al.* 2017 | |
| VP103 | *h- smto* | | *pcf1::kanMX6, rtf1:nmt41:sup35, t>ura4^+^<ori, ade6-704, leu1-32* | | Pietrobon *et al*. 2014 | |
| VP247 | *h- smto* | | *pcf1-YFP:kanMX6, rtf1::nmt41:sup35, t>ura4^+^<ori, ade6-704, leu1-32* | | This study | |
| VP285 | *h+* | | *pcf1::ura4*^+^, *ade6-704, leu1-32, ura4-D18* | | Pietrobon *et al*. 2014 | |
| VP316 | *h- smto* | | *rqh1::kanMX6, pcf1::ura4*^+^, *ade6-704, leu1-32, ura4-D18* | | Pietrobon *et al*. 2014 | |
| VP394 | *h- smto* | | *pcf1-YFP:kanMX6, ade6-704, leu1-32, ura4-D18* | | Pietrobon *et al*. 2014 | |
| VP426 | *h- smto* | | *pcf1-YFP:kanMX6, pcf2-4.5-MYC:kanMX6, ade6-704, leu1-32, ura4-D18* | | Pietrobon *et al*. 2014 | |
| VP453 | *h-* | | *hht1-hhf1::his3,^+^ hht3-hhf3::arg3^+^, rtf1:nmt41:sup35, t>ura4+<ori, leu1-32, ade6-704, ade6-otr, his3-D1, arg3-D4* | | This study | |
| VP464 | *h-* | | *hip1::kanMX6, ade6-704, leu1-32, ura4-D18* | | This study | |
| VP467 | *h+* | | *hip1::kanMX6, ade6-704, leu1-32, ura4-D18* | | This study | |
| VP476 | *h+* | | *rtt109::kanMX6, ade6-M216, leu1-32, ura4-D18* | | Bioneer | |
| VP478 | *h+* | | *hht1-hhf1::his3^+^, hht3-hhf3::arg3^+^, hht2-K56R, rtf1:nmt41:sup35, t>ura4^+^<ori, leu1-32, ade6-210, ade6-otr, his3-D1, arg3-D4* | | This study | |
| VP480 | *h+* | | *rtt109::kanMX6, rtf1:nmt41:sup35, t>ura4^+^<ori, ade6-704, leu1-32* | | This study | |
| VP481 | *h-* | | *slm9::kanMX6, rtf1:nmt41:sup35, t>ura4^+^<ori, ade6-704, leu1-32* | | This study | |
| VP484 | *h+* | | *asf1-MYC:kanMX6, rtf1:nmt41:sup35, t>ura4^+^<ori, ade6-704, leu1-32* | | This study | |
| VP486 | *h- smto* | | *asf1-33-MYC:kanMX6, rtf1:nmt41:sup35, t>ura4^+^<ori, ade6-704, leu1-32* | | This study | |
| VP488 | *h+* | | *rtt106::kanMX6, rtf1:nmt41:sup35, t>ura4^+^<ori, ade6-704, leu1-32* | | This study | |
| VP490 | *h+* | | *nap1::kanMX6, rtf1:nmt41:sup35, t>ura4^+^<ori, ade6-704, leu1-32* | | This study | |
| VP511 | *h- smto* | | *hht2-H113D:hphMX, rtf1:nmt41:sup35, t>ura4^+^<ori, ade6-704, leu1-32* | | This study | |
| VP520 | *h- smto* | | *rqh1-MYC:kanMX6, pcf1-YFP:kanMX6, ura4-D18, ade6-704, leu1-32* | | Pietrobon *et al*. 2014 | |
| VP543 | *h+* | | *hht2-H113D:hphMX, rtf1:nmt41:sup35, t>ura4^+^<ori, ade6-704, leu1-32* | | This study | |
| VP562 | *h-smto* | | *hip1::kanMX6, rtf1:nmt41:sup35, t>ura4^+^<ori, ade6-704, leu1-32* | | This study | |
