# Supplementary material for: Histone deposition promotes recombination-dependent replication at arrested forks
Source: PLoS Genet. 2019 Oct 4;15(10):e1008441. doi: 10.1371/journal.pgen.1008441 (PMC6795475; doi:10.1371/journal.pgen.1008441)
Supplement: S2 Table — (DOCX) [file pgen.1008441.s007.docx]

**S2 Table:** **List of primers used in this study**

| Name | Sequence (5’-3’) | Experiment |
| --- | --- | --- |
| #1 (R400F) | CACACTTGCTCTGTACACGTATTCT | ChIP Pcf1-YFP and Rad52 |
| #1 (R400R) | AGGATCCATGATGCACAGATT | ChIP Pcf1-YFP and Rad52 |
| #2 (R5F) | TTGCCAAACATCCTCCTACC | ChIP Rad52 |
| #2 (R5R) | GAAACACAAGCCAAAGTTGC | ChIP Rad52 |
| #3 (Ura4-1F) | GACTCCACGACCAACAATGA | ChIP Pcf1-YFP and Rad52 |
| #3 (Ura4-1R) | CTGGTATCGGCTTGGATGTT | ChIP Pcf1-YFP and Rad52 |
| #4 (L3F) | TTTAAATCAAATCTTCCATGCG | ChIP Pcf1-YFP and Rad52 |
| #4 (L3R) | TGTACCCATGAGCAAACTGC | ChIP Pcf1-YFP and Rad52 |
| #5 (L400F) | ATCTGACATGGCATTCCTCA | ChIP Rad52 |
| #5 (L400R) | GATGCCAGACCGTAATGACA | ChIP Rad52 |
| #6 (L600F) | CCATTGACTAGGAGGACTTTGAG | ChIP Rad52 |
| #6 (L600R) | CCCTGGCGGTTGTAGTTAGT | ChIP Rad52 |
| Ade6-23 | GGCTGCCTCTACCATCATTC | ChIP Pcf1-YFP and Rad52 |
| Ade6-25 | TTAAGCTGAGCTGCCAAGGT | ChIP Pcf1-YFP and Rad52 |
| RuraR probe 1F | CAAACGCAAACAAGGCATCGAC | ^32^p probe |
| RuraR probe 1R | GGCTCTTTGGCTACTGGTTC | ^32^p probe |
| RNG3 lo | AAGGACTGCGTTCTTCTAGC | ^32^p probe |
| RNG3 up | TGAATCCTCCGTTCAGTAGG | ^32^p probe |
